# Targeting multiple pro-apoptotic signaling pathways with curcumin in prostate cancer cells

**DOI:** 10.1371/journal.pone.0179587

**Published:** 2017-06-19

**Authors:** Mariela Rivera, Yanilda Ramos, Madeline Rodríguez-Valentín, Sheila López-Acevedo, Luis A. Cubano, Jin Zou, Qiang Zhang, Guangdi Wang, Nawal M. Boukli

**Affiliations:** 1 Department of Microbiology and Immunology, Biomedical Proteomics Facility, Universidad Central del Caribe School of Medicine, Bayamón, Puerto Rico, United States of America; 2 Center for Cancer Research and Therapeutic Development, Clark Atlanta University, Atlanta, Georgia, United States of America; 3 Department of Chemistry, RCMI Cancer Research Center, Xavier University of Louisiana, New Orleans, Louisiana, United States of America; University of South Alabama Mitchell Cancer Institute, UNITED STATES

## Abstract

Curcumin, an extract from the turmeric rhizome (*Curcuma longa*), is known to exhibit anti-inflammatory, antioxidant, chemopreventive and antitumoral activities against aggressive and recurrent cancers. Accumulative data indicate that curcumin may induce cancer cell death. However, the detailed mechanism underlying its pro-apoptotic and anti-cancer effects remains to be elucidated. In the present study, we examined the signaling pathways triggered by curcumin, specifically, the exact molecular mechanisms of curcumin-induced apoptosis in highly metastatic human prostate cancer cells. The effect of curcumin was evaluated using for the first time in prostate cancer, a gel-free shotgun quantitative proteomic analysis coupled with Tandem Mass Tag isobaric labeling-based-signaling networks. Results were confirmed at the gene expression level by qRT-PCR and at the protein expression level by western blot and flow cytometry. Our findings revealed that curcumin induced an Endoplasmic Reticulum stress-mediated apoptosis in PC3. The mechanisms by which curcumin promoted cell death in these cells were associated with cell cycle arrest, increased reactive oxygen species, autophagy and the Unfolded Protein Response. Furthermore, the upregulation of ER stress was measured using key indicators of ER stress: Glucose-Regulated Protein 78, Inositol-Requiring Enzyme 1 alpha, Protein Disulfide isomerase and Calreticulin. Chronic ER stress induction was concomitant with the upregulation of pro-apoptotic markers (caspases 3,9,12) and Poly (ADP-ribose) polymerase. The downregulated proteins include anti-apoptotic and anti-tumor markers, supporting their curcumin-induced pro-apoptotic role in prostate cancer cells. Taken together, these data suggest that curcumin may serve as a promising anticancer agent by inducing a chronic ER stress mediated cell death and activation of cell cycle arrest, UPR, autophagy and oxidative stress responses.

## Introduction

Prostate cancer (Pca) is one of the most common malignancies and the second leading cause of tumor-related mortality in American males [1]. Conventional therapies produce a high rate of cure for patients with localized prostate cancer by surgical therapy, but there is no cure once the disease has spread beyond the prostate. In spite of advances in chemotherapy, Pca therapies have not been completely successful in eradicating the disease and have been found to be toxic to normal patient cells [2], thus, new agents are needed to selectively kill cancer cells. For the past several centuries, natural products/dietary phytochemical compounds have been used in the treatment of cancer and are still the major source of new drug development. Among nontoxic natural compounds, curcumin, the active ingredient in *Curcuma longa*, is known for its ability to target cancer cells and for its therapeutic and anti-invasive properties [3–5]. Curcumin is a natural phenolic compound that has been studied for its anti-proliferative, anti-inflammatory, and anti-oxidative properties [6, 7]. The purpose of the present study was to investigate the effects of curcumin on highly metastatic human prostate cancer cells (PC3) and the mechanisms underlying its anticancer and anti-apoptotic properties. We speculate that curcumin alters the expression of proteins associated with pro-apoptotic mechanisms, one of them being chronic ER stress mediated cell death. When the cell undergoes cytotoxic conditions, protein misfolding occurs, leading to unfolded proteins aggregating in the ER. To correct misfolding, the cell triggers the unfolded protein response (UPR). Chaperones and folding enzymes, such as the 78-kD Glucose-Regulated Protein (GRP78), protein disulfide isomerase (PDI), eukaryotic translation initiation factor 2 alpha (eIF2α), and calreticulin (CALR) have been shown to play a key role in this process. Under acute ER stress conditions, the cell resorts to survival, while chronic ER stress triggers cell death. The microenvironments of cancerous cells have been shown to exert ER stress resistance, which is required for the survival and growth of tumors. However, prolonged or severe ER stress may ultimately overwhelm the cellular protective mechanisms, triggering cell death through specific programmed cell death pathways [8]. Apoptosis prompted by chronic ER stress can occur through the intrinsic pathway, which is activated by the transcription factor CCAAT-enhancer-binding protein homologous protein (CHOP) and subsequent caspase activation [3].

UPR and ER stress may induce the activation of alternative stress pathways such as autophagy, reactive oxygen species (ROS) responses, cell cycle arrest, among others [9]. Autophagy is initiated by encapsulation of cytoplasmic components (proteins and organelles) within isolation membranes to form autophagosomes. These structures eventually fuse with lysosomes and the cargo is degraded. Accumulative data indicated that the UPR may induce autophagy and that it can alleviate the UPR or promote cell death [10, 11]. However, the detailed mechanism of interplay between autophagy and UPR remains to be fully understood. Activation of the double-stranded RNA-activated protein kinase (PKR)-like endoplasmic reticulum (ER) kinase (PERK)–eukaryotic translation initiation factor 2 alpha (eIF2α)–activating transcription factor 4 (ATF4) pathway upregulates the expression of a large set of autophagy genes. Functionally, autophagy promotes cell survival, increases energy supply, and mediates innate immune responses. Nevertheless, autophagy can also be associated with cell death and apoptosis, depending on the cell stress level.

Reactive Oxygen Species (ROS) is referred to oxygen containing reactive molecules and free radicals derived from metabolism of oxygen and is responsible for causing significant damages in the cell, increasing the intracellular amount of mitochondrial biogenesis or responses. These elevated ROS mediate adverse consequences in cells and have been linked to ER stress and the UPR [12]. In a stressed ER, dysregulated disulfide bond formation and breakage may result in reactive oxygen species (ROS) accumulation and cause oxidative stress [13]. This is followed by Ca^2+^ release from the ER and mitochondrial ROS production creating a vicious cycle that impairs cellular homeostasis and induces apoptosis. Given the deleterious impact of oxidative stress induced during protein misfolding in the ER, eukaryotic cells have evolved antioxidative stress responses to restore cellular redox homeostasis. The PERK branch of the UPR induces antioxidative stress response genes, and is responsible for uncoupling mitochondrial protein 2 [14]. In settings of chronic and prolonged ER stress conditions, these mechanisms may lead to apoptosis induction [15]. The results obtained in the present study indicate that curcumin induced a chronic ER stress mediated cell death, and activated an orchestrated mechanism associated with cell cycle arrest, UPR, autophagy and oxidative stress response.

The identification of key differentially expressed proteins has been successfully applied to understand curcumin’s effect on PCa by means of a gel-free shotgun quantitative proteomic analysis coupled with Tandem Mass Tag Isobaric Labeling. The results were validated using qRT-PCR, flow cytometry and western blotting. Results from these analyses provide insights into curcumin mechanisms and suggest that curcumin induced specifically a chronic ER stress mediated cell death in PC3. We speculate that curcumin induces apoptosis in PC3 by activating cell cycle arrest, oxidative stress and UPR.

## Materials and methods

### Cell culture

The PC3 cell line (ATCC) was derived from a bone metastasis from a grade IV prostatic adenocarcinoma patient. PC3 cells were cultured in complete F-12K medium supplemented with 10% fetal bovine serum (FBS, Invitrogen, Grand Island, NY) and 1% penicillin/streptomycin (Invitrogen) at a density of 5 x 10^4^ cells/cm^2^ in 75cm^2^ culture flasks (BD Biosciences). Cells were maintained in a cell culture incubator at 37°C in a humidified atmosphere with 5% CO_2_.

### Prostate cancer cell (PC3) treatment with curcumin

Approximately 2 x 10^6^ PC3 cells were seeded in 75-cm^2^ culture flasks in supplemented F-12K medium, allowed to adhere for 24 hours, and maintained in a humidified atmosphere with 5% CO_2_ and 37°C. Curcumin was added at a concentration of 5 μg/ml, as previously published in collaboration with Kurapati et al. [16]. This concentration was chosen since it showed significant inhibition of colony formation in clonogenic assays at 5 μg/mL in PC3 cells. PC3 cells were incubated for 72 hours while control cells were treated with 0.4% (by volume) dimethyl sulfoxide (DMSO, Sigma-Aldrich, St. Louis, MO) for 72 hours as previously described [17]. Curcumin was obtained from Sigma Aldrich and dissolved in DMSO at a concentration of 1mg/ml.

### Acridine orange analysis

Acridine orange (Sigma Aldrich) was used to assess autophagy. Cells were seeded in 25cm^2^ flasks and treated with curcumin or DMSO for three days, cells were washed with PBS and stained with 5μg/ml of acridine orange. Images were acquired on an Olympus IX70 inverted fluorescent microscope (Olympus America Inc., Center Valley, NJ), using the Q Capture Pro software (QImaging, Surrey, Canada).

### Cell viability by ReadiDrop 7-AAD

7-aminoactinomycin D (BioRad, Hercules CA), was used to determine cell viability after treatment with either curcumin or DMSO. The manufacturer’s protocol was followed. Stained cells were measured by flow cytometry with a FACSCanto II (Becton Dickinson, San Jose, CA, USA). Data analysis was performed using FlowJo data analysis software v. 10 (Ashland, OR).

### Gel-free isobaric labeling tandem mass tag quantitative proteomic profiling of PC3 cells treated with curcumin

#### Cell lysis and protein extraction

Cells were plated on 150 cm^2^ culture plates at a cell density of 5 × 10^6^ and treated the following day with 5 μg/ml curcumin for 72 h. Cells treated with DMSO were used as controls. Proteins were extracted with RIPA (1.5 M Tris pH 8.8, 1.75 g NaCl, 2 mL sodium dodecyl sulfate 10%, 2 mL Triton X-100; all reagents from Thermo Fisher Scientific, (Waltham, MA). The cells were incubated on ice for 30 min, followed by 5 min sonication and centrifugation at 20,000 rpm for 5 min in preparation for protein extraction. Protein concentration was calculated on microtiter plates by measuring the absorbance at 595 nm of samples containing Bradford reagent (Bio-Rad Laboratories, Hercules, CA) supplemented with phosphatase and protease inhibitor cocktail (Roche Molecular Biochemicals, Indianapolis, IN).

#### Reduction, alkylation, and trypsin digestion

Aliquots with 100 mg of proteins from each sample were added to 100 ml of 200 mM triethyl ammonium bicarbonate TEAB (Sigma-Aldrich, St. Louis, MO). Reduction was performed by adding 5 ml of 200 mM tris (2-carboxyethyl) phosphine TCEP (Sigma-Aldrich, St. Louis, MO) to each replicate and incubating for 1 h at 55°C. Alkylation was carried out by adding 5 ml of 375 mM iodoacetamide (Bio-Rad Laboratories, Hercules, CA) to each sample and incubating for 30 min at room temperature. After alkylation, 1 ml of pre-chilled acetone was added and precipitation was allowed to proceed for 3 h at 20°C. Acetone-precipitated protein pellets were suspended in 100 ml of 200 mM TEAB and digested overnight at 37°C with 2.5 μg of sequencing grade modified trypsin (Promega Corp., Madison, WI) as previously described [18].

#### Isobaric labeling with tandem mass tag

The non-gel isobaric labeling Tandem Mass Tag (TMT) quantitative proteomic approach was performed as previously described in Burton et al, 2016 [18]. TMTs with varying molecular weights (126–131 Da) (Thermo Fisher Scientific, Waltham, MA) were used as isobaric labels to determine differential protein expression between PC3 control cells treated with DMSO (0.4%) and PC3 cells treated with 5μg/ml curcumin. According to the manufacturer’s protocols, the six digested samples were individually labeled with TMT reagents as follows. Three control (DMSO-treated) samples, TMT-126 (batch 1), TMT-127 (batch 2), and TMT-128 (batch 3); and three curcumin-treated samples, TMT-129 (batch 1), TMT-130 (batch 2), and TMT-131 (batch 3). The labeled peptide mixtures were combined in equal ratios. The TMT-labeled peptide mixture was fractionated with a strong cation exchange SCX column (Thermo Fisher Scientific, Waltham, MA) on a Shimadzu 2010 high performance liquid chromatography (HPLC) equipped with an ultraviolet detector (Shimadzu, Columbia, MD) and a mobile phase consisting of buffer A (5 mM KH_2_PO_4_, 25% acetonitrile, pH 2.8) and buffer B (buffer A plus 350 mM KCl). The column was equilibrated with buffer A for 30 min before sample injection. A mobile phase gradient at a flow rate of 1.0 ml/min was set as follows: 1) 0 ~ 10 min: 0% buffer B; 2) 10 ~ 40 min: 0 ~ 25% buffer B; 3) 40 ~ 45 min: 25 ~ 100% buffer B; 4) 45 ~ 50 min: 100% buffer B; 5) 50 ~ 60 min: 100 ~ 0% buffer B; and 6) 60 ~ 90 min: 0% buffer B. Sixty fractions were initially collected, lyophilized, and combined into 10 final fractions based on SCX chromatogram peaks.

#### Desalination of fractionated samples

A C_18_ solid phase extraction SPE column (Thermo Fisher Scientific, Waltham, MA) was used to desalt all collected fractions as previously described. Briefly, the 10 combined fractions were each adjusted to a final volume of 1 ml in a 0.25% trifluoroacetic acid (TFA) aqueous solution. The C_18_ SPE column was preconditioned with 1 ml acetonitrile and eluted in approximately 3 min before it was rinsed with 3 × 1 ml 0.25% TFA. The fractions were loaded on to the top of the SPE cartridge column slowly, and were reloaded once again to decrease lost peptide during column binding. Columns were washed with 4 × 1 ml 0.25% TFA aliquots before the peptides were eluted with 3 × 400 μl 80% acetonitrile/0.1% formic acid (aqueous). The eluted samples were lyophilized prior to the liquid chromatography mass spectrometry LC-MS/MS analysis.

#### LC-MS/MS analysis

Peptides were analyzed on an LTQ-Orbitrap XL (Thermo Fisher Scientific, Waltham, MA) instrument interfaced with an Ultimate 3000 Dionex LC system (Dionex, Sunnyvale, CA). High mass resolution was utilized for peptide identification and high-energy collision dissociation (HCD) was used for reporter ion quantification as previously described [18]. Briefly, the reverse phase LC system contained a peptide Cap-Trap cartridge (0.5 × 2 mm) (Michrom BioResources, Auburn, CA) and a pre-packed BioBasic C_18_ PicoFrit analytical column (75 μm i.d. × 15 cm length, New Objective, Woburn, MA) fitted with a FortisTip emitter tip. Samples were loaded onto the trap cartridge and washed with mobile phase A (98% H_2_O, 2% acetonitrile, and 0.1% formic acid) for concentration and desalting. Peptides were eluted over 180 min from the analytical column via the trap cartridge by using a linear gradient of 6 to 100% mobile phase B (20% H_2_O, 80% acetonitrile, and 0.1% formic acid) at a flow rate of 0.3 μl/min. The Orbitrap mass spectrometer was operated in a data-dependent mode in which each full MS scan (60,000 resolving power) was followed by six MS/MS scans where the three most abundant molecular ions were dynamically selected and fragmented by collision-induced dissociation with a normalized collision energy of 35% and subsequently scanned by HCD-MS^2^ with a collision energy of 45% as previously described [18]. Only the 2+, 3+, and 4+ ions were selected for fragmentation by collision-induced dissociation and HCD.

#### Database search and TMT quantification

The protein search algorithm SEQUEST was used to identify unique protein peptides using the Proteome Discoverer data processing software (version 1.2, Thermo Fisher Scientific, Waltham, MA). The ratios of TMT reporter ion abundances in MS/MS spectra generated by HCD from raw data sets were used For TMT quantification. Fold changes in proteins between control and treatment were calculated as previously described [18].

### Intracellular staining by flow cytometry

Cells were blocked for 10 minutes with 1% of BSA/PBS and incubated for 30 minutes in ice with the use of the rabbit polyclonal antibody against human cleaved caspase 3 (1:800) for experiments assessing cell death and p-eIF2α (1:100) for experiments assessing translational attenuation (Cell Signaling Technology, Danvers, MA). Cells were acquired and analyzed on a FACSCanto II and FlowJo software (TreeStar, Ashland, OR).

### Cleaved caspase-3 sandwich ELISA

Quantification of cleaved caspase-3 was performed using the PathScan Cleaved Caspase-3 (Asp175) sandwich ELISA kit (Cell Signaling) according to manufacturer’s recommendations.

### Intracellular oxidative species

Intracellular oxidation levels were determined by 2',7'-dichlorodihydrofluorescein diacetate staining (H2DCFDA; Molecular Probes, Eugene, OR) which in the presence of intracellular ROS is oxidized to highly fluorescent 2′,7′-dichlorofluorescein (DCF). After treatment with curcumin or DMSO, cells were incubated at 37°C for 30 minutes with 1μM of HD2CFDA. The dye was removed and cells were washed once with PBS and analyzed on a FACSCanto II and FlowJo software (TreeStar, Ashland, OR).

### Cell cycle analysis

For cell cycle assays, 1 × 10^6^ curcumin treated PC3 and control cells were fixed with 70% ethanol solution at 4°C for 1 hour, then washed with 1X PBS, and incubated with a 0.2 mg/mL RNase A (Sigma Aldrich, St. Lois, MO) for 1 h at 37°C. The cell suspension was supplemented with 10 μg/mL of propidium iodide (Sigma Aldrich, St. Lois, MO) and incubated at 4°C for 1 hour protected from light. Samples were analyzed on a FACSCanto II and FlowJo software (TreeStar, Ashland, OR).

### Western blot

Proteins were extracted with RIPA buffer (1.5 M Tris pH 8.8, 1.75 g NaCl, 2 mL of 10% sodium dodecyl sulfate, 2 mL Triton X-100; all reagents from Fisher Scientific) plus a protease inhibitor cocktail. Protein quantification was performed with the Pierce™ BCA Protein Assay Kit (Thermo Fisher Scientific, Waltham, MA). The samples absorbance was read and analyzed using the SoftMax Pro software (Molecular Devices, Sunnyvale, CA) coupled to a VersaMax Tunable microplate reader (Molecular Devices, Sunnyvale, CA) detecting at 562 nm excitation. Proteins were then analyzed by SDS-PAGE and transferred to a PVDF membrane. Membranes were blocked with 5% non-fat dry milk in Tris-buffered saline and Tween 20 (TBST) and incubated with primary antibodies overnight at 4°C. The primary antibodies used in this experiment were: anti-casp 12 (1:1000 dilution; Abcam, Cambridge, MA), anti-Ire1α (1:1000, Cell Signaling Technology), anti-GRP78 (1:1000, Cell Signaling Technology), anti-calreticulin (1:1000, Abcam), anti-PARP (1:1000, Cell Signaling Technology) and anti-LC3B (1:1000, Cell Signaling Technology). To confirm equal loading, blots were stained with 0.1% India ink. Horseradish peroxidase-conjugated secondary antibodies were used, and protein bands were visualized by a chemiluminescent method using the SuperSignal West Femto Maximum Sensitivity Substrate kit (Thermo Fisher Scientific, Waltham, MA). Images were analyzed with the ImageJ software (Bethesda, MD, USA) by comparing band densities (peak percent).

### Quantitative real-time PCR

SYBR green qRT-PCR gene expression assays was performed for Caspase-3 (*CASP3*) and Caspase-9 (*CASP9*) genes. Amplification was carried out in a Bio-Rad CFX96 Touch real-time PCR detection system (Bio-Rad, Hercules, CA) using the following program: 10 minutes at 95°C, 50 cycles of 15 seconds at 95°C, and 1 minute at the gene-specific annealing temperature. The gene-specific primers were: *CASP-3* forward 5’-AAATGAATGGGCTGAGCTGC-3’; *CASP-3* reverse 5’-GCGTATGGAGAAATGGGCTG-3’; CASP-9 forward 5’-AAGTGACCCTCCCAAGTAGC-3’; CASP-9 reverse 5’-GAGACCTAACCTCCCTGAGC-3’. The gene expression level was defined as the threshold cycle number (CT). Mean fold changes in expression of the target genes were calculated using the comparative CT method (RU, 2–ΔΔCt). All data were controlled for the quantity of RNA input by *18S* forward 5’-GGCCCTGTAATTGGAATGAGTC-3’; *18S* reverse 5’-CCAAGATCCAACTACGAGCTT -3’ serving as the endogenous control and for normalization.

### Statistical analysis

Cytoxicity assays, quantitative PCR and proteomics experiments were performed in biological and technical triplicates, using separate trials with similar results. Statistical analysis was performed using two-way ANOVA and unpaired t-tests, as appropriate, by means of GraphPad PRISM v6.03 statistical software (GraphPad Software, La Jolla, CA). p<0.05 was considered statistically significant.

## Results

### Proteomic profiling of PC3 cells treated with curcumin: A TMT isobaric-labeling quantitative gel free proteomic approach

The differential expression of proteins between untreated (0.4% DMSO) and treated (5 μg/ml curcumin) PC3 cells was determined based upon an isobaric labeling, TMT, quantitative proteomic approach for further validation and identification of novel proteins. Comparative quantitative proteomics identified over 926 proteins (S1 Table) in control and treated PC3 cell lysates, out of which 330 proteins were differentially expressed. Proteins with a statistically significant fold change ≥ 1.2 or ≤ -1.2-fold were considered differentially expressed. The detailed information including gene symbol, gene name, fold change, p value, molecular weight and calculated pI are shown in Table 1. Because it was not feasible to discuss all identified proteins (926), the selection criteria were based on significance in terms of fold change.

**Table 1:** Overexpressed proteins identified in PC3 cells treated with curcumin and arranged in decreasing fold change order.

**Upregulated Proteins**					
***Gene Symbol***	***Protein Name***	***Fold Change***	***P value***	***MW (kDa)***	***pI***
***SLC1A5***	solute carrier family 1 (neutral amino acid transporter), member 5	2.03	5.69E-05	56.56	5.48
***TBC1D10B***	TBC1 domain family, member 10B	1.93	1.41E-06	87.14	9.19
***MRPS17***	mitochondrial ribosomal protein S17	1.91	1.94E-04	14.49	9.85
***YBX1***	Y box binding protein 1	1.75	2.27E-05	34.65	7.53
***HSPA5***	heat shock 70kDa protein 5 (GRP78)	1.69	4.98E-07	72.29	5.16
***DAK***	dihydroxyacetone kinase 2 homolog (S. cerevisiae)	1.61	1.12E-01	58.91	7.49
***PRPF40A***	PRP40 pre-mRNA processing factor 40 homolog A (S. cerevisiae)	1.61	1.53E-04	95.04	7.36
***EEF1G***	eukaryotic translation elongation factor 1 gamma	1.59	8.06E-05	24.09	7.05
***SRSF1***	serine/arginine-rich splicing factor 1	1.59	2.45E-03	22.45	7.94
***YARS***	tyrosyl-tRNA synthetase	1.59	1.29E-05	59.11	7.05
***SLC3A2***	solute carrier family 3 (amino acid transporter heavy chain), member 2	1.57	1.93E-04	55.9	5.17
***SNRPF***	small nuclear ribonucleoprotein polypeptide F	1.56	6.28E-03	9.72	4.67
***HIST1H4A***	Histone H4	1.53	6.66E-05	11.36	11.4
***HSPE1***	heat shock 10kDa protein 1 (chaperonin 10)	1.51	3.48E-06	10.92	8.92
***EFTUD2***	elongation factor Tu GTP binding domain containing 2	1.5	3.80E-03	105.32	5.22
***SF3B1***	splicing factor 3b, subunit 1, 155kDa	1.5	9.97E-04	145.74	7.09
***APOL1***	apolipoprotein L, 1	1.49	2.20E-08	18.1	5.43
***HSP90B1***	heat shock protein 90kDa beta (Grp94), member 1	1.48	2.54E-05	92.41	4.84
***PLCH1***	phospholipase C, eta 1	1.48	2.44E-07	114.43	7.64
***ABHD10***	abhydrolase domain containing 10	1.47	1.31E-05	16.9	6.73
***ANXA1***	annexin A1	1.45	2.12E-05	38.69	7.02
***LAMP2***	lysosomal-associated membrane protein 2	1.45	7.05E-04	39.79	5.67
***STC2***	stanniocalcin 2	1.45	2.22E-03	33.23	7.3
***GRPEL1***	GrpE-like 1, mitochondrial (E. coli)	1.44	2.61E-05	21.89	8.62
***HNRNPAB***	heterogeneous nuclear ribonucleoprotein A/B	1.44	1.69E-05	30.28	7.91
***TPD52L2***	tumor protein D52-like 2	1.44	1.73E-05	19.89	5.48
***CYB5B***	cytochrome b5 type B (outer mitochondrial membrane)	1.42	1.28E-02	14.84	6.19
***DDX17***	DEAD (Asp-Glu-Ala-Asp) box helicase 17	1.42	4.12E-02	47.53	7.66
***KPNA3***	karyopherin alpha 3 (importin alpha 4)	1.42	8.36E-02	10	3.95
***AGR2***	anterior gradient 2	1.4	1.77E-04	19.97	9
***ANXA7***	annexin A7	1.4	8.45E-05	37.78	7.18
***EEF1E1***	eukaryotic translation elongation factor 1 epsilon 1	1.4	3.45E-03	5.88	8.62
***FUS***	fused in sarcoma	1.4	2.26E-05	15.23	9.41
***FLNB***	filamin B, beta	1.39	1.44E-04	275.5	5.78
***KHSRP***	KH-type splicing regulatory protein	1.39	1.06E-04	72.85	7.9
***ALYREF***	Aly/REF export factor	1.38	1.12E-04	26.87	11.2
***CLTA***	clathrin, light chain A	1.38	3.94E-03	23.65	4.53
***SRXN1***	sulfiredoxin 1	1.38	5.85E-02	14.25	8.19
***ACADVL***	acyl-CoA dehydrogenase, very long chain	1.37	2.27E-01	68.02	8.56
***PSAP***	prosaposin	1.37	2.61E-05	50.4	5.01
***SNRPD1***	small nuclear ribonucleoprotein D1 polypeptide 16kDa	1.37	5.63E-06	13.27	11.6
***KRT75***	keratin 75	1.36	3.20E-03	59.52	7.74
***S100A6***	S100 calcium binding protein A6	1.36	7.20E-06	10.17	5.48
***EEF1B2***	eukaryotic translation elongation factor 1 beta 2	1.35	6.96E-05	3.1	4.46
***RPS19***	ribosomal protein S19	1.35	7.13E-05	16.05	10.3
***HNRNPA2B1***	heterogeneous nuclear ribonucleoprotein A2/B1	1.34	8.97E-05	35.98	8.65
***SNRPA***	small nuclear ribonucleoprotein polypeptide A	1.34	5.79E-04	31.26	9.83
***AHSG***	alpha-2-HS-glycoprotein	1.33	2.63E-03	39.3	5.72
***HIST1H2BL***	histone cluster 1, H2bl	1.33	7.96E-05	13.94	10.3
***PGRMC1***	progesterone receptor membrane component 1	1.33	6.83E-05	21.66	4.7
***SNRPD3***	small nuclear ribonucleoprotein D3 polypeptide 18kDa	1.33	1.02E-05	13.28	8.91
***SRP54***	signal recognition particle 54kDa	1.33	5.66E-04	48.64	9.04
***VCPIP1***	valosin containing protein (p97)/p47 complex interacting protein 1	1.33	3.16E-02	134.24	7.2
***CLTC***	clathrin, heavy chain (Hc)	1.32	3.65E-08	187.77	5.69
***GOLGB1***	golgin B1	1.32	1.39E-01	375.79	5
***HNRNPD***	heterogeneous nuclear ribonucleoprotein D (AU-rich element RNA binding protein 1, 37kDa)	1.32	2.65E-05	12.63	8.57
***NES***	nestin	1.32	2.46E-07	177.33	4.36
***POR***	P450 (cytochrome) oxidoreductase	1.32	1.28E-02	47.37	7.81
***PDIA6***	protein disulfide isomerase family A, member 6	1.31	1.87E-04	47.81	5.08
***RPS21***	ribosomal protein S21	1.31	1.07E-03	8.84	8.5
***SNRPD2***	small nuclear ribonucleoprotein D2 polypeptide 16.5kDa	1.31	1.55E-04	12.39	9.89
***STOML2***	stomatin (EPB72)-like 2	1.31	6.46E-05	33.32	8.25
***GCSH***	glycine cleavage system protein H (aminomethyl carrier)	1.3	1.71E-04	18.87	4.88
***PRKCSH***	protein kinase C substrate 80K-H	1.3	6.04E-05	59.14	4.42
***RPS16***	ribosomal protein S16	1.3	1.21E-03	16.44	10.2
***S100P***	S100 calcium binding protein P	1.3	2.54E-02	10.39	4.88
***TMED2***	transmembrane emp24 domain trafficking protein 2	1.3	4.88E-03	17.07	4.84
***ALB***	albumin	1.29	1.40E-03	51.54	6.95
***ANXA11***	annexin A11	1.29	2.84E-05	45.7	8.46
***EIF3K***	eukaryotic translation initiation factor 3, subunit K	1.29	6.97E-02	25.04	4.93
***FAU***	Finkel-Biskis-Reilly murine sarcoma virus (FBR-MuSV) ubiquitously expressed	1.29	1.32E-05	10.9	11.6
***LETM1***	leucine zipper-EF-hand containing transmembrane protein 1	1.29	7.20E-04	83.3	6.7
***PDAP1***	PDGFA associated protein 1	1.29	1.15E-02	4.39	10.3
***PKM***	pyruvate kinase, muscle	1.29	1.33E-01	58.02	7.71
***PRRC1***	proline-rich coiled-coil 1	1.29	5.39E-04	46.67	5.83
***RAP1B***	RAP1B, member of RAS oncogene family	1.29	9.51E-06	18.77	8.53
***RPS10***	ribosomal protein S10	1.29	1.19E-05	18.89	10.2
***SERBP1***	SERPINE1 mRNA binding protein 1	1.29	3.70E-04	42.4	8.44
***BSG***	basigin (Ok blood group)	1.28	2.34E-02	19.43	6.68
***LAMTOR2***	late endosomal/lysosomal adaptor, MAPK and MTOR activator 2	1.28	2.04E-03	13.5	5.4
***NOLC1***	nucleolar and coiled-body phosphoprotein 1	1.28	8.75E-04	73.56	9.47
***PDIA3***	protein disulfide isomerase family A, member 3	1.28	1.89E-06	54.07	7.21
***PLCB3***	phospholipase C, beta 3 (phosphatidylinositol-specific)	1.28	2.37E-06	131.12	5.88
***PPIB***	peptidylprolyl isomerase B (cyclophilin B)	1.28	5.59E-04	23.73	9.41
***PSMB2***	proteasome (prosome, macropain) subunit, beta type, 2	1.28	7.64E-04	9.55	8.78
***SRSF2***	serine/arginine-rich splicing factor 2	1.28	1.44E-03	23.18	11.8
***CD59***	CD59 molecule, complement regulatory protein	1.27	2.57E-01	11.98	5.74
***ERP29***	endoplasmic reticulum protein 29	1.27	1.57E-06	5.47	9.72
***PDIA4***	protein disulfide isomerase family A, member 4	1.27	5.23E-05	72.89	5.07
***TFRC***	transferrin receptor	1.27	5.69E-05	75.92	6.81
***TPD52***	tumor protein D52	1.27	6.66E-02	12.38	4.68
***CTNNBL1***	catenin, beta like 1	1.26	1.20E-01	36.08	6.7
***DBN1***	drebrin 1	1.26	1.75E-03	36.45	5.14
***GNG12***	guanine nucleotide binding protein (G protein), gamma 12	1.26	2.68E-02	8	8.97
***HRSP12***	heat-responsive protein 12	1.26	3.05E-02	11.82	8.76
***NPM1***	nucleophosmin (nucleolar phosphoprotein B23, numatrin)	1.26	1.40E-05	29.45	4.61
***OGDH***	oxoglutarate (alpha-ketoglutarate) dehydrogenase (lipoamide)	1.26	3.90E-06	98.95	7.46
***RPS11***	ribosomal protein S11	1.26	2.43E-04	18.42	10.3
***VIM***	vimentin	1.26	1.15E-05	53.62	5.12
***ACOT8***	acyl-CoA thioesterase 8	1.25	3.28E-01	9.91	5.71
***API5***	apoptosis inhibitor 5	1.25	9.53E-05	48.66	5.19
***EIF2A***	eukaryotic translation initiation factor 2A, 65kDa	1.25	1.35E-01	35.28	8.5
***HNRNPD***	heterogeneous nuclear ribonucleoprotein D (AU-rich element RNA binding protein 1, 37kDa)	1.25	5.93E-03	23.06	5.11
***PDIA3***	protein disulfide isomerase family A, member 3	1.25	7.72E-03	13.51	7.3
***PPP1R16A***	protein phosphatase 1, regulatory subunit 16A	1.25	2.84E-04	57.78	6.06
***ANXA5***	annexin A5	1.24	4.54E-06	35.91	5.05
***MRPL12***	Mitochondrial Ribosomal Protein L12	1.24	3.62E-04	21.33	8.87
***NAA15***	N(alpha)-acetyltransferase 15, NatA auxiliary subunit	1.24	5.55E-04	86.39	6.35
***PDLIM1***	PDZ and LIM domain 1	1.24	1.55E-02	36.05	7.02
***POFUT1***	protein O-fucosyltransferase 1	1.24	1.82E-04	22.32	8.76
***RAP2B***	RAP2B, member of RAS oncogene family	1.24	1.31E-03	20.49	4.81
***RPS28***	ribosomal protein S28	1.24	7.82E-04	7.84	10.7
***VPS35***	vacuolar protein sorting 35 homolog (S. cerevisiae)	1.24	2.40E-01	76.16	5.43
***HNRNPA1***	heterogeneous nuclear ribonucleoprotein A1	1.23	1.01E-03	29.37	9.14
***IDH1***	isocitrate dehydrogenase 1 (NADP+), soluble	1.23	3.48E-01	46.63	7.01
***MAP4***	microtubule-associated protein 4	1.23	2.08E-03	120.93	5.43
***RPL13***	ribosomal protein L13	1.23	5.07E-04	24.25	11.7
***RPS12***	ribosomal protein S12	1.23	5.47E-05	14.51	7.21
***RPS4X***	ribosomal protein S4, X-linked	1.23	3.86E-04	29.58	10.2
***EIF2S2***	Eukaryotic translation initiation factor 2 subunit 2	1.22	5.59E-05	37.76	6.34
***EIF5A***	eukaryotic translation initiation factor 5A	1.22	6.80E-06	16.82	5.24
***MYOF***	myoferlin	1.22	6.17E-06	179.44	6.33
***PFDN4***	prefoldin subunit 4	1.22	2.21E-01	15.3	4.53
***PKM***	pyruvate kinase, muscle	1.22	6.68E-07	57.9	7.84
***PRDX3***	peroxiredoxin 3	1.22	1.59E-04	25.82	7.46
***RPL27***	ribosomal protein L27	1.22	7.66E-03	15.79	10.6
***RPL36***	ribosomal protein L36	1.22	6.74E-03	12.25	11.6
***SCP2***	sterol carrier protein 2	1.22	9.60E-03	13.03	5.57
***SRP14***	signal recognition particle 14kDa (homologous Alu RNA binding protein)	1.22	2.09E-04	14.56	10
***SYNCRIP***	synaptotagmin binding, cytoplasmic RNA interacting protein	1.22	1.06E-04	58.7	7.56
***ALDH7A1***	aldehyde dehydrogenase 7 family, member A1	1.21	6.51E-02	55.33	6.86
***FH***	fumarate hydratase	1.21	2.84E-05	50.18	7.4
***HNRNPK***	heterogeneous nuclear ribonucleoprotein K	1.21	1.03E-04	47.53	5.63
***UBAP2L***	ubiquitin associated protein 2-like	1.21	1.59E-01	103.11	6.87
***AHNAK***	AHNAK nucleoprotein	1.2	1.31E-05	628.7	6.15
***ATP5G1***	ATP synthase, H+ transporting, mitochondrial Fo complex, subunit C1 (subunit 9)	1.2	5.48E-01	10.19	10.4
***CAB39***	calcium binding protein 39	1.2	1.46E-02	39.84	6.89
***DDX1***	DEAD (Asp-Glu-Ala-Asp) box helicase 1	1.2	4.27E-03	69.51	7.72
***NACA***	nascent polypeptide-associated complex alpha subunit	1.2	2.37E-04	15.01	4.91
***RPS25***	ribosomal protein S25	1.2	2.44E-04	13.73	10.1
***S100A11***	S100 calcium binding protein A11	1.2	3.27E-04	11.73	7.12
***SRP9***	signal recognition particle 9kDa	1.2	6.10E-04	10.11	7.97
***CANX***	calnexin	1.19	5.10E-05	67.53	4.6
***CD55***	CD55 molecule, decay accelerating factor for complement (Cromer blood group)	1.19	6.49E-04	41.2	8.03
***HSPA9***	heat shock 70kDa protein 9 (mortalin)	1.19	1.32E-05	72.36	5.94
***MTAP***	methylthioadenosine phosphorylase	1.19	2.26E-03	33.16	7.46
**Downregulated Proteins**					
***Gene Symbol***	***Protein Name***	***Fold Change***	***P value***	***MW (kDa)***	***pI***
***CDK1***	cyclin-dependent kinase 1	-3.81	9.17E-05	27.49	7.06
***PUF60***	poly-U binding splicing factor 60KDa	-2.57	1.67E-02	26.93	8.81
***NONO***	non-POU domain containing, octamer-binding	-2.19	4.72E-02	23.66	9.8
***HNRNPM***	Heterogeneous nuclear ribonucleoprotein M	-1.95	4.40E-07	39.16	9.14
***TUFM***	Tu translation elongation factor, mitochondrial	-1.9	1.28E-03	49.84	7.61
***ADK***	adenosine kinase	-1.88	6.77E-05	38.68	6.68
***ARL1***	ADP-ribosylation factor-like 1	-1.86	1.92E-06	5.84	6.62
***OSGEP***	O-sialoglycoprotein endopeptidase	-1.84	1.60E-03	15.94	8.05
***ARF1***	ADP-ribosylation factor 1	-1.83	6.67E-04	20.68	6.8
***HNRNPU***	heterogeneous nuclear ribonucleoprotein U (scaffold attachment factor A)	-1.82	5.15E-06	83.03	8.79
***PTGES3***	prostaglandin E synthase 3 (cytosolic)	-1.78	1.04E-05	14.83	4.11
***NSUN2***	NOP2/Sun RNA methyltransferase family, member 2	-1.72	6.96E-04	59.35	6.55
***LOC100508782***	inositol hexakisphosphate and diphosphoinositol-pentakisphosphate kinase 1-like	-1.69	3.13E-03	91.02	7.78
***TPI1***	Triosephosphate isomerase	-1.68	6.05E-06	26.65	6.9
***CLIC4***	chloride intracellular channel 4	-1.67	8.88E-04	23.25	5.34
***ENO1***	enolase 1, (alpha)	-1.67	7.57E-03	36.9	6.28
***WDR61***	WD repeat domain 61	-1.64	2.26E-04	33.56	5.47
***ARHGDIA***	Rho GDP dissociation inhibitor (GDI) alpha	-1.62	2.26E-02	18.22	5.19
***OTUB1***	OTU domain, ubiquitin aldehyde binding 1	-1.62	1.67E-05	28.03	5.29
***CMPK1***	cytidine monophosphate (UMP-CMP) kinase 1, cytosolic	-1.61	1.48E-05	20.55	8.95
***PRKAG1***	protein kinase, AMP-activated, gamma 1 non-catalytic subunit	-1.6	1.30E-02	28.27	7.02
***ARF4***	ADP-ribosylation factor 4	-1.59	1.87E-05	20.5	7.14
***EEF1A1***	eukaryotic translation elongation factor 1 alpha 1	-1.59	4.05E-04	50.11	9.01
***FDPS***	farnesyl diphosphate synthase	-1.59	8.46E-05	39.63	4.98
***GSTM3***	glutathione S-transferase mu 3 (brain)	-1.59	5.81E-06	24.8	5.24
***UBA2***	ubiquitin-like modifier activating enzyme 2	-1.57	4.22E-03	60.8	5.07
***SAE1***	SUMO1 activating enzyme subunit 1	-1.56	5.85E-04	19.14	5.31
***LRPPRC***	leucine-rich pentatricopeptide repeat containing	-1.55	4.65E-05	123.2	5.54
***P4HA1***	prolyl 4-hydroxylase, alpha polypeptide I	-1.54	1.77E-05	58.92	6.02
***LTA4H***	leukotriene A4 hydrolase	-1.53	1.01E-03	57.26	6.06
***COPS2***	COP9 signalosome subunit 2	-1.52	1.10E-02	44.2	5.87
***KIAA0196***	KIAA0196	-1.52	1.95E-01	116.99	8.27
***NDRG1***	N-myc downstream regulated 1	-1.52	4.81E-05	38.55	6.34
***APRT***	adenine phosphoribosyltransferase	-1.51	6.44E-05	19.6	6.02
***EIF4A3***	eukaryotic translation initiation factor 4A3	-1.51	2.25E-04	46.84	6.73
***NOL3***	nucleolar protein 3 (apoptosis repressor with CARD domain)	-1.51	1.26E-02	24.31	11.3
***PARK7***	protein DJ-1	-1.51	8.34E-07	19.88	6.79
***EIF6***	eukaryotic translation initiation factor 6	-1.5	2.02E-03	26.58	4.68
***PCBP2***	poly(rC) binding protein 2	-1.5	2.82E-05	16.99	7.69
***CAMK2A***	calcium/calmodulin-dependent protein kinase II alpha	-1.48	4.93E-04	2.56	9.72
***PMPCA***	peptidase (mitochondrial processing) alpha	-1.48	3.66E-03	28.22	6.76
***PSMC6***	proteasome (prosome, macropain) 26S subunit, ATPase, 6	-1.47	3.61E-04	8.7	6.11
***ALDH1A3***	aldehyde dehydrogenase 1 family, member A3	-1.46	1.45E-03	45.41	8.18
***LGALS1***	lectin, galactoside-binding, soluble, 1	-1.46	2.15E-05	14.71	5.5
***TUBB***	tubulin, beta class I	-1.46	8.42E-06	47.74	4.81
***ALDH18A1***	aldehyde dehydrogenase 18 family, member A1	-1.45	1.25E-02	87.03	7.12
***KCNH3***	potassium voltage-gated channel, subfamily H (eag-related), member 3	-1.45	9.78E-05	117.05	7.94
***PPP2R4***	protein phosphatase 2A activator, regulatory subunit 4	-1.45	1.09E-02	14.42	5.96
***RAN***	RAs-related Nuclear protein	-1.45	2.67E-05	24.41	7.49
***RAB3IP***	RAB3A interacting protein	-1.44	2.31E-04	10.27	4.68
***PFN1***	profilin 1	-1.43	1.09E-06	15.04	8.27
***PPP1R14B***	protein phosphatase 1, regulatory (inhibitor) subunit 14B	-1.43	1.02E-04	20.95	5.22
***TUBB4B***	tubulin, beta 4B class IVb	-1.43	3.74E-05	49.8	4.89
***AP3D1***	adaptor-related protein complex 3, delta 1 subunit	-1.42	1.91E-01	111.17	8.1
***DDB1***	damage-specific DNA binding protein 1, 127kDa	-1.42	2.08E-03	14.27	5.27
***GAPDH***	glyceraldehyde-3-phosphate dehydrogenase	-1.42	5.34E-06	36.03	8.46
***HSP90AA1***	heat shock protein 90kDa alpha (cytosolic), class A member 1	-1.42	2.19E-08	84.61	5.02
***IL18***	interleukin 18 (interferon-gamma-inducing factor)	-1.42	1.01E-02	22.31	4.67
***MGST1***	microsomal glutathione S-transferase 1	-1.42	4.37E-02	4.84	6.51
***PGAM1***	Phosphoglycerate Mutase 1	-1.42	3.58E-06	27.08	8.24
***PGK1***	phosphoglycerate kinase 1	-1.42	2.17E-05	41.4	7.33
***RRM1***	ribonucleotide reductase M1	-1.42	2.35E-03	86.4	6.65
***SH3BGRL3***	SH3 domain binding glutamic acid-rich protein like 3	-1.42	1.48E-03	9.37	9.36
***SLC25A3***	solute carrier family 25 (mitochondrial carrier; phosphate carrier), member 3	-1.42	1.68E-02	16.94	8.47
***TALDO1***	transaldolase 1	-1.42	7.90E-06	37.52	6.81
***TUBA4A***	tubulin, alpha 4a	-1.42	2.77E-05	48.3	5.01
***DSTN***	destrin (actin depolymerizing factor)	-1.41	3.16E-04	15.39	8.59
***GCN1L1***	GCN1 general control of amino-acid synthesis 1-like 1 (yeast)	-1.41	1.06E-03	292.57	7.47
***TRIM28***	tripartite motif containing 28	-1.41	3.46E-04	79.42	5.99
***VAPA***	VAMP (vesicle-associated membrane protein)-associated protein A, 33kDa	-1.4	3.24E-04	27.88	8.62
***GSTP1***	glutathione S-transferase pi 1	-1.39	1.12E-04	19.47	5.97
***PRDX5***	peroxiredoxin 5	-1.39	2.03E-06	17.02	7.24
***PRDX6***	peroxiredoxin 6	-1.39	1.85E-05	25.02	6.38
***ENO1***	enolase 1, (alpha)	-1.38	3.04E-07	47.14	7.39
***NQO1***	NAD(P)H dehydrogenase, quinone 1	-1.38	3.89E-02	22.78	8.5
***PSMC1***	proteasome (prosome, macropain) 26S subunit, ATPase, 1	-1.38	2.44E-04	41.14	5.01
***TUBB4A***	tubulin, beta 4A class IVa	-1.38	5.72E-03	49.55	4.88
***UBA1***	ubiquitin-like modifier activating enzyme 1	-1.38	8.03E-06	117.77	5.76
***ALDH1B1***	aldehyde dehydrogenase 1 family, member B1	-1.37	4.26E-05	57.17	6.8
***HADHA***	hydroxyacyl-CoA dehydrogenase/3-ketoacyl-CoA thiolase/enoyl-CoA hydratase (trifunctional protein), alpha subunit	-1.37	8.28E-05	82.95	9.04
***SRPR***	signal recognition particle receptor (docking protein)	-1.37	1.09E-02	66.52	9.04
***TUBA1B***	tubulin, α1b	-1.37	3.11E-05	50.12	5.06
***FXR1***	fragile X mental retardation, autosomal homolog 1	-1.36	2.66E-03	50.96	7.27
***COTL1***	coactosin-like 1 (Dictyostelium)	-1.35	3.16E-04	15.94	5.67
***DDX21***	DEAD (Asp-Glu-Ala-Asp) box helicase 21	-1.35	1.82E-02	79.61	9.38
***GLO1***	glyoxalase I	-1.35	3.27E-03	19.03	6.05
***HUWE1***	HECT, UBA and WWE domain containing 1, E3 ubiquitin protein ligase	-1.35	2.91E-01	186.1	5.97
***PPP1CA***	protein phosphatase 1, catalytic subunit, alpha isozyme	-1.35	4.48E-06	28.88	4.87
***BTF3***	basic transcription factor 3	-1.34	2.36E-02	9.38	5
***CALCRL***	calcitonin receptor-like	-1.34	4.58E-01	54.28	6.77
***CSE1L***	CSE1 chromosome segregation 1-like (yeast)	-1.34	7.39E-05	107.71	5.82
***FKBP1A***	FK506 binding protein 1A, 12kDa	-1.34	9.10E-02	3.96	5.78
***HSP90AB1***	heat shock protein 90kDa alpha (cytosolic), class B member 1	-1.34	2.94E-06	83.21	5.03
***PFKP***	phosphofructokinase, platelet	-1.34	2.27E-03	22.66	5.2
***PTMS***	Parathymosin	-1.34	8.71E-03	6.25	8.12
***ALDH4A1***	aldehyde dehydrogenase 4 family, member A1	-1.33	7.10E-02	21.21	6.79
***ALDOC***	aldolase C, fructose-bisphosphate	-1.33	1.56E-04	48.38	8.07
***NANS***	N-acetylneuraminic acid synthase	-1.33	4.75E-03	40.28	6.74
***PGM1***	phosphoglucomutase 1	-1.33	2.79E-02	58.73	5.95
***SFN***	Stratifin	-1.33	6.47E-05	27.76	4.74
***SKP1***	S-phase kinase-associated protein 1	-1.33	1.80E-02	16.02	4.64
***UBC***	ubiquitin C	-1.33	2.23E-05	16.83	6.58
***VDAC2***	voltage-dependent anion channel 2	-1.33	4.75E-06	27.46	5.47
***FABP5***	fatty acid binding protein 5 (psoriasis-associated)	-1.32	3.47E-05	15.15	7.01
***GART***	Trifunctional purine biosynthetic protein adenosine-3	-1.32	8.71E-05	46	6.79
***HADHB***	hydroxyacyl-CoA dehydrogenase/3-ketoacyl-CoA thiolase/enoyl-CoA hydratase (trifunctional protein), beta subunit	-1.32	9.00E-04	40.39	9.79
***HPRT1***	hypoxanthine phosphoribosyltransferase 1	-1.32	1.60E-04	24.56	6.68
***MYH9***	myosin, heavy chain 9, non-muscle	-1.32	2.13E-05	226.39	5.6
***PAFAH1B2***	platelet-activating factor acetylhydrolase 1b, catalytic subunit 2 (30kDa)	-1.32	3.85E-03	18.35	6.27
***SCCPDH***	saccharopine dehydrogenase (putative)	-1.32	5.15E-01	47.12	9.14
***SPAG9***	sperm associated antigen 9	-1.32	1.58E-04	54.36	4.64
***AK1***	adenylate kinase 1	-1.31	1.24E-03	21.62	8.63
***CBX1***	chromobox homolog 1	-1.31	6.41E-05	19.31	5.02
***EIF4A1***	eukaryotic translation initiation factor 4A1	-1.31	4.59E-05	23.24	5.69
***HSD17B10***	hydroxysteroid (17-beta) dehydrogenase 10	-1.31	2.04E-05	26.91	7.78
***PEBP1***	phosphatidylethanolamine binding protein 1	-1.31	9.00E-05	21.04	7.53
***ALDOA***	aldolase A, fructose-bisphosphate	-1.3	3.37E-07	39.4	8.09
***DDX3X***	DEAD (Asp-Glu-Ala-Asp) box helicase 3, X-linked	-1.3	3.03E-05	71.31	6.62
***DLAT***	dihydrolipoamide S-acetyltransferase	-1.3	5.41E-04	44.57	6.2
***IARS2***	isoleucyl-tRNA synthetase 2, mitochondrial	-1.3	7.99E-03	113.72	7.2
***COPS7B***	COP9 signalosome subunit 7B	-1.29	8.77E-04	16.95	4.86
***DNM1L***	dynamin 1-like	-1.29	5.48E-03	64.14	7.9
***EIF4A1***	eukaryotic translation initiation factor 4A1	-1.29	4.34E-04	46.12	5.48
***IMPDH2***	IMP (inosine 5'-monophosphate) dehydrogenase 2	-1.29	3.89E-02	55.77	6.9
***PSMC4***	proteasome (prosome, macropain) 26S subunit, ATPase, 4	-1.29	9.56E-06	43.48	5.26
***SAR1A***	SAR1 homolog A (S. cerevisiae)	-1.29	5.71E-05	22.35	6.68
***TLR9***	toll-like receptor 9	-1.29	1.34E-04	6.27	8.5
***TTLL3***	tubulin tyrosine ligase-like family, member 3	-1.29	4.88E-02	49.38	8.91
***UBE2M***	ubiquitin-conjugating enzyme E2M	-1.29	5.04E-04	20.89	7.69
***CPNE3***	copine III	-1.28	4.64E-04	60.09	5.85
***GTPBP3***	GTP binding protein 3 (mitochondrial)	-1.28	7.61E-04	49.82	6.8
***PDLIM5***	PDZ and LIM domain 5	-1.28	3.05E-04	48.93	7.87
***SERPINB5***	serpin peptidase inhibitor, clade B (ovalbumin), member 5	-1.28	3.26E-01	14.35	5.57
***YWHAG***	tyrosine 3-monooxygenase/tryptophan 5-monooxygenase activation protein, gamma polypeptide	-1.28	6.45E-04	28.28	4.89
***ACOT9***	acyl-CoA thioesterase 9	-1.27	3.29E-02	15.45	8.59
***AKAP12***	A kinase (PRKA) anchor protein 12	-1.27	1.45E-03	180.88	4.44
***ATP2A2***	ATPase, Ca++ transporting, cardiac muscle, slow twitch 2	-1.27	7.93E-03	109.62	5.36
***CNDP2***	CNDP dipeptidase 2 (metallopeptidase M20 family)	-1.27	9.58E-03	30.36	5.66
***DHPS***	deoxyhypusine synthase	-1.27	4.56E-03	15.08	5
***EIF4H***	eukaryotic translation initiation factor 4H	-1.27	3.13E-02	27.37	7.23
***ERO1L***	ERO1-like (S. cerevisiae)	-1.27	8.67E-04	8.82	8.69
***HSDL2***	hydroxysteroid dehydrogenase like 2	-1.27	6.73E-03	37.3	6.1
***NASP***	nuclear autoantigenic sperm protein (histone-binding)	-1.27	1.31E-02	28.18	4.23
***PSMD2***	proteasome (prosome, macropain) 26S subunit, non-ATPase, 2	-1.27	1.01E-03	99.25	5.16
***RCN2***	reticulocalbin 2, EF-hand calcium binding domain	-1.27	1.32E-01	36.85	4.4
***GDI2***	GDP dissociation inhibitor 2	-1.26	7.38E-05	50.63	6.47
***KIF22***	kinesin family member 22	-1.26	2.25E-04	66.27	8.91
***PNP***	purine nucleoside phosphorylase	-1.26	7.00E-04	32.53	7.21
***PRKAR1A***	protein kinase, cAMP-dependent, regulatory, type I, alpha	-1.26	2.72E-01	42.95	5.35
***PSMC3***	proteasome (prosome, macropain) 26S subunit, ATPase, 3	-1.26	7.16E-03	19.89	4.91
***RNH1***	ribonuclease/angiogenin inhibitor 1	-1.26	1.54E-02	20.46	4.73
***UBE2L3***	ubiquitin-conjugating enzyme E2L 3	-1.26	9.61E-03	14.11	8.92
***KPNB1***	karyopherin (importin) beta 1	-1.25	2.29E-04	97.11	4.78
***MAP1S***	microtubule-associated protein 1S	-1.25	3.68E-01	109.72	7.65
***PPIA***	peptidylprolyl isomerase A (cyclophilin A)	-1.25	3.34E-05	18	7.81
***PSMD5***	proteasome (prosome, macropain) 26S subunit, non-ATPase, 5	-1.25	1.49E-02	56.16	5.48
***PPA1***	pyrophosphatase (inorganic) 1	-1.24	7.99E-06	32.64	5.86
***PYGL***	phosphorylase, glycogen, liver	-1.24	3.78E-03	93.08	7.3
***INF2***	inverted formin, FH2 and WH2 domain containing	-1.23	8.21E-03	134.53	5.53
***SSB***	Sjogren syndrome antigen B (autoantigen La)	-1.23	7.67E-05	41.8	5.96
***TIAM1***	T-cell lymphoma invasion and metastasis 1	-1.23	1.45E-03	118.58	6.15
***TRMT1***	tRNA methyltransferase 1 homolog (S. cerevisiae)	-1.23	1.74E-02	69.26	7.23
***XPO1***	exportin 1 (CRM1 homolog, yeast)	-1.23	6.39E-06	123.31	6.06
***HSPH1***	heat shock 105kDa/110kDa protein 1	-1.22	5.64E-04	77.11	5.1
***MIF***	macrophage migration inhibitory factor (glycosylation-inhibiting factor)	-1.22	3.48E-04	12.47	7.88
***PHB***	Prohibitin	-1.22	3.65E-03	29.79	5.76
***RPN1***	ribophorin I	-1.22	1.53E-04	72.73	6.28
***RPS15***	ribosomal protein S15	-1.22	1.43E-03	17.03	10.4
***SRM***	spermidine synthase	-1.22	1.67E-03	33.8	5.49
***YWHAQ***	tyrosine 3-monooxygenase/tryptophan 5-monooxygenase activation protein, theta polypeptide	-1.22	2.54E-05	27.75	4.78
***YWHAZ***	tyrosine 3-monooxygenase/tryptophan 5-monooxygenase activation protein, zeta polypeptide	-1.22	1.33E-05	27.73	4.79
***ACO2***	aconitase 2, mitochondrial	-1.21	1.35E-02	54.43	7.62
***ATP9A***	ATPase, class II, type 9A	-1.21	1.95E-01	105.07	8.16
***PPP2R1A***	protein phosphatase 2, regulatory subunit A, alpha	-1.21	6.24E-06	61.48	5.12
***TLN1***	talin 1	-1.21	1.86E-02	257.92	6.49
***CAND1***	cullin-associated and neddylation-dissociated 1	-1.2	9.40E-04	136.29	5.78
***ETFB***	electron-transfer-flavoprotein, beta polypeptide	-1.2	1.47E-04	27.83	8.1
***FKBP4***	FK506 binding protein 4, 59kDa	-1.2	4.27E-03	51.77	5.43
***IQGAP1***	IQ motif containing GTPase activating protein 1	-1.2	5.20E-03	189.13	6.48
***MDH1***	malate dehydrogenase 1, NAD (soluble)	-1.2	1.63E-02	36.4	7.36
***MRPL9***	mitochondrial ribosomal protein L9	-1.2	4.20E-01	17.48	10.7
***MYL6***	myosin, light chain 6, alkali, smooth muscle and non-muscle	-1.2	2.52E-03	11.67	4.41
***NUDT21***	nudix (nucleoside diphosphate linked moiety X)-type motif 21	-1.2	2.57E-01	26.21	8.82
***SMS***	spermine synthase	-1.2	8.92E-04	31.08	4.93

MW—Molecular Weight (kDa)

IP—Isoelectric Point

Negative numbers—downregulated; Positive numbers–upregulated

Proteins with a statistically significant fold change ≥ 1.2 or ≤ -1.2-fold were considered differentially expressed.

Curcumin-treated PC3 cells expressed 146 upregulated and 184 downregulated proteins when compared with control PC3 cells (treated with DMSO). Of the identified proteins upregulated by curcumin figure ER stress markers such as GRP78 (Glucose Regulated Protein 78kDa or HSPA5) and PDI. These two proteins need special mention since they are involved in sensing and respond to the accumulation of unfolded or misfolded proteins in the ER. GRP78 and the PDI family were upregulated by 1.69 and ≥1.25-fold respectively as determined by the TMT 6-plex quantitation method. Curcumin treatment led to the upregulation of several ER stress markers and chaperones such as HSPA9 and ERP29 with significant fold change levels of 1.19 and 1.27, respectively in PC3 cells. Among other upregulated proteins related to ER stress figure eukaryotic translation initiation factor 2A (EIF2A), with a significant fold change of 1.25, and the ER stress pro-apoptotic protein calnexin (CANX) [19], which was significantly increased to 1.19-fold relative expression in the curcumin-treatment group as compared to control (*p ≤ 0.05; *n = 3*) (Table 1). The differentially expressed proteins included downregulated antioxidant markers such as peroxiredoxin 6 (PRDX6) and protein DJ-1 (PARK7) with significant fold changes of –1.39 and –1.51, respectively, correlating with the upregulation of reactive oxygen species markers such as annexin A1 (ANXA1) [20] and sulfiredoxin-1 (SRXN1), with fold changes of 1.45 and 1.38, respectively, in curcumin-treated PC3 cells. Furthermore, curcumin altered the expression of pro-apoptotic markers (annexin A5, PGRMC1 and vimentin), anti-apoptotic protein signatures (Inorganic pyrophosphatase and VAMP-associated protein A); cell cycle, migration and proliferation proteins (cyclin-dependent kinase 1, heat shock protein HSP 90-alpha and beta, endoplasmic reticulum protein 29, Ras-related protein Rap-2b, PPP1CA); endoplasmic-reticulum-associated degradation (ERAD) activation (PMPCA, PSMB2, PSMC1, PSMD2); oxidative stress regulators (PARK7, PDZ and LIM domain protein 1, sulfiredoxin); cytoskeleton and transport proteins (vimentin, profilin, myosin, dynamin, destrin, filamin B, tubulin and transmembrane emp24) and autophagy related proteins (Rab GDP dissociation inhibitor beta, protein DJ-1 and ubiquitin-like modifier-activating enzyme 1) in PC3 cells.

### Curcumin extract reduces cell viability and induces a cell cycle arrest in PC3 cells

**Fig 1:**
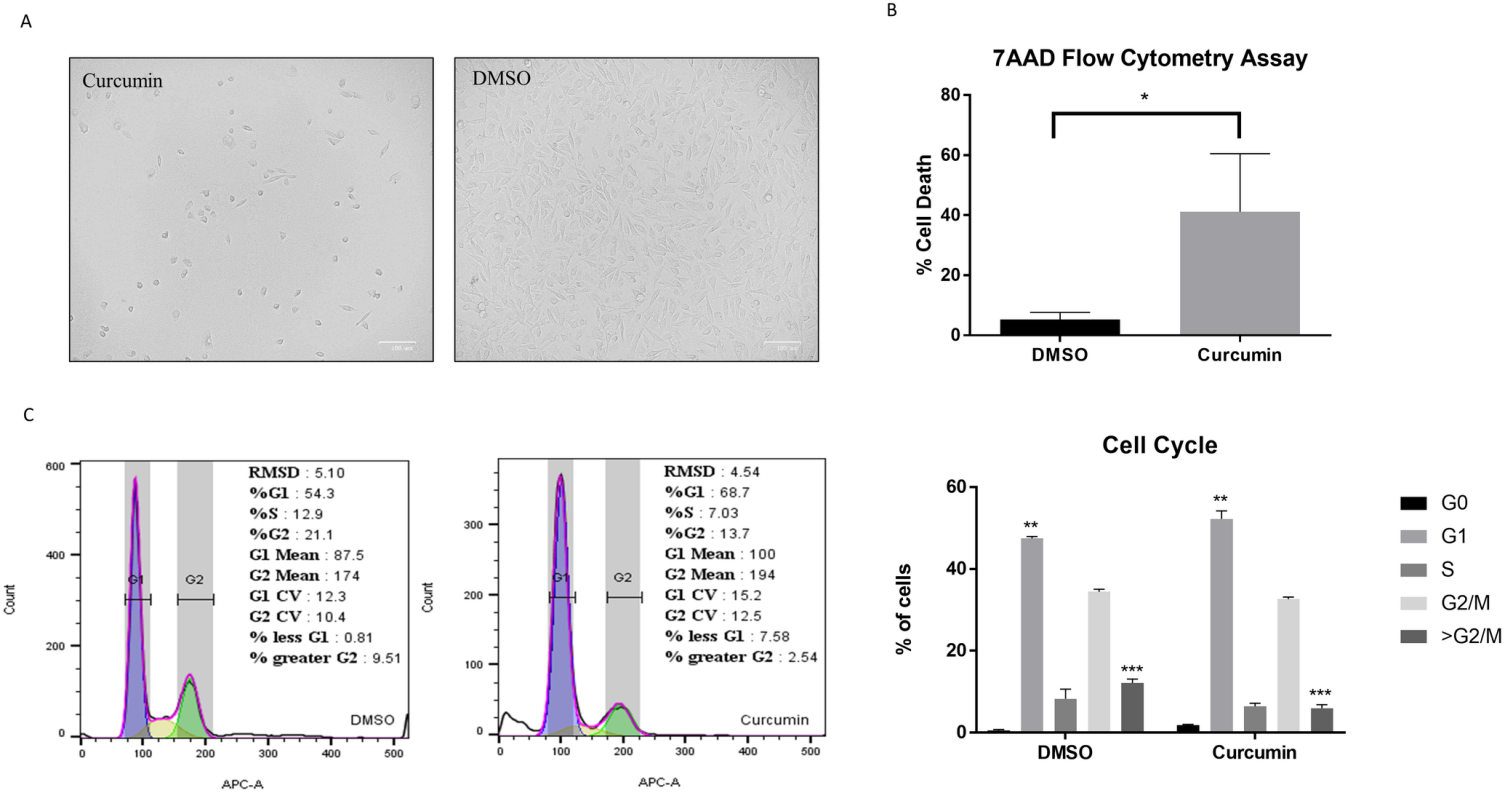
Curcumin inhibits cell proliferation and promotes cell death. (A) Optical micrograph of PC3 confluency after treatment with either Curcumin or DMSO. (B) Percentage of death cells stained with 7AAD, analyzed by flow cytometry and compared by unpaired t-test, p≤0.05. (C) Cell cycle analysis by flow cytometry; statistical analysis was determined by Two-way ANOVA, *p≤0.05, **p≤0.01.

As previously reported by Kurapati et al. [16], *C*. *longa* showed significant inhibition of colony formation in clonogenic assays at 5 μg/mL in PC3 cells, a dose we chose in our assays. The confluency of the PC3 cell line was evaluated for changes in response to treatment with curcumin compared to DMSO. At 72 hrs, cells treated with 5 μg/ml of curcumin diminished their confluency when compared to DMSO (Fig 1A). To further evaluate the cytotoxicity of curcumin extract in PC3, a 7AAD assay was performed. Our results confirmed that curcumin induces approximately 40% of cell death vs 5% in DMSO (Fig 1B, p value ≤ 0.03). We evaluated the cell cycle effect induced by curcumin in PC3 cells, since the quantitative TMT proteomic profiling revealed differentially expressed cell cycle proteins. Cell cycle analysis revealed that curcumin treatment induced a cell cycle arrest at the G1 phase. The percentage of cells arrested in G1 was significantly higher in curcumin than DMSO (Fig 1C, p value ≤ 0.0020). The G0 peak was also increased under curcumin treatment and the percent of cells greater than G2/M was significantly higher in DMSO (p value ≤ 0.0002). These results suggest that curcumin not only induces a cytotoxic effect in PC3 cells but can also deregulate the cell cycle by promoting a G0/G1 arrest.

### Curcumin induces the upregulation of pro-apoptotic markers in PC3 cells

**Fig 2:**
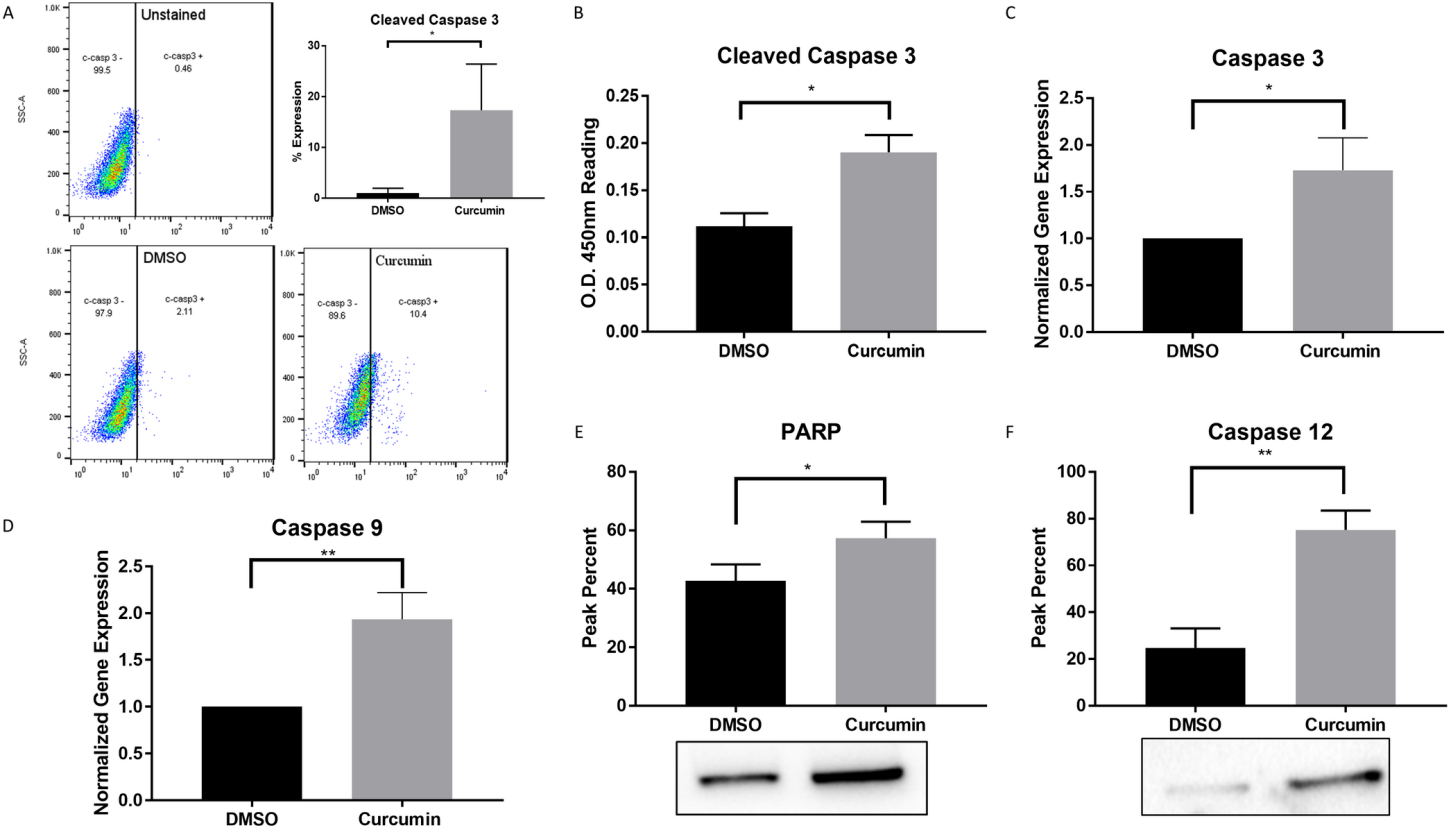
Curcumin induces caspase-mediated apoptosis. (A) Cleaved caspase 3 protein expression determined by flow cytometry. (B) Validation of cleaved caspase 3 protein expression by PathScan Sandwich ELISA. (C) Uncleaved caspase 3 gene expression analyzed by qRT-PCR. (D-F) Caspase 9, PARP and Caspase 12 protein expressions assessed by western blotting. Statistical significance between Curcumin and DMSO was determined by unpaired t-test, * p≤0.05, ** p≤0.01.

To confirm the apoptotic curcumin-induced protein alterations obtained by the quantitative proteomic results (Table 1), caspase dependent pro-apoptotic expression was evaluated to assess other cell death signaling mechanisms. Protein expression of cleaved caspase 3, an apoptotic effector protein, was evaluated using flow cytometry analysis. Approximately 17% of cells treated with curcumin exhibited cleaved caspase 3 expression when compared to 1% in DMSO (Fig 2A, p value ≤ 0.036). To validate the flow cytometry data, an ELISA assay on cells treated with curcumin or DMSO was assessed. Curcumin treated cells exhibited higher expression of cleaved caspase 3 when compared to DMSO (Fig 2B). The un-cleaved expression of caspase 3 was evaluated by qRT-PCR with a result of nearly 1.7-fold vs 1.0 in DMSO and a p-value ≤ 0.021 (Fig 2C). Caspase 9 activity was measured as a caspase initiator and upstream processor of effector caspase 3 with further apoptotic propagation. Curcumin treated cells showed an increase of 1.93-fold over DMSO (Fig 2D). Correspondingly, Poly (ADP-ribose) polymerase (PARP), a programmed cell death effector, had significantly higher expression upon curcumin treatment when compared to DMSO by means of western blot (Fig 2E, p value ≤ 0.0107). In order to further correlate the quantitative proteomic data, caspase 12 expression, a central player in ER stress induced apoptosis and cytotoxicity [21] was evaluated. Curcumin PC3 treated cells induced a significantly higher expression of caspase 12 when compared to DMSO, with a peak percent in the range of 75% vs. 25% in DMSO (Fig 2F, p value ≤ 0.0017), suggesting that curcumin triggers a chronic ER stress induced cell death in prostate cancer cells.

### Curcumin-induced the up regulation of ER stress markers in PC3 cells

**Fig 3:**
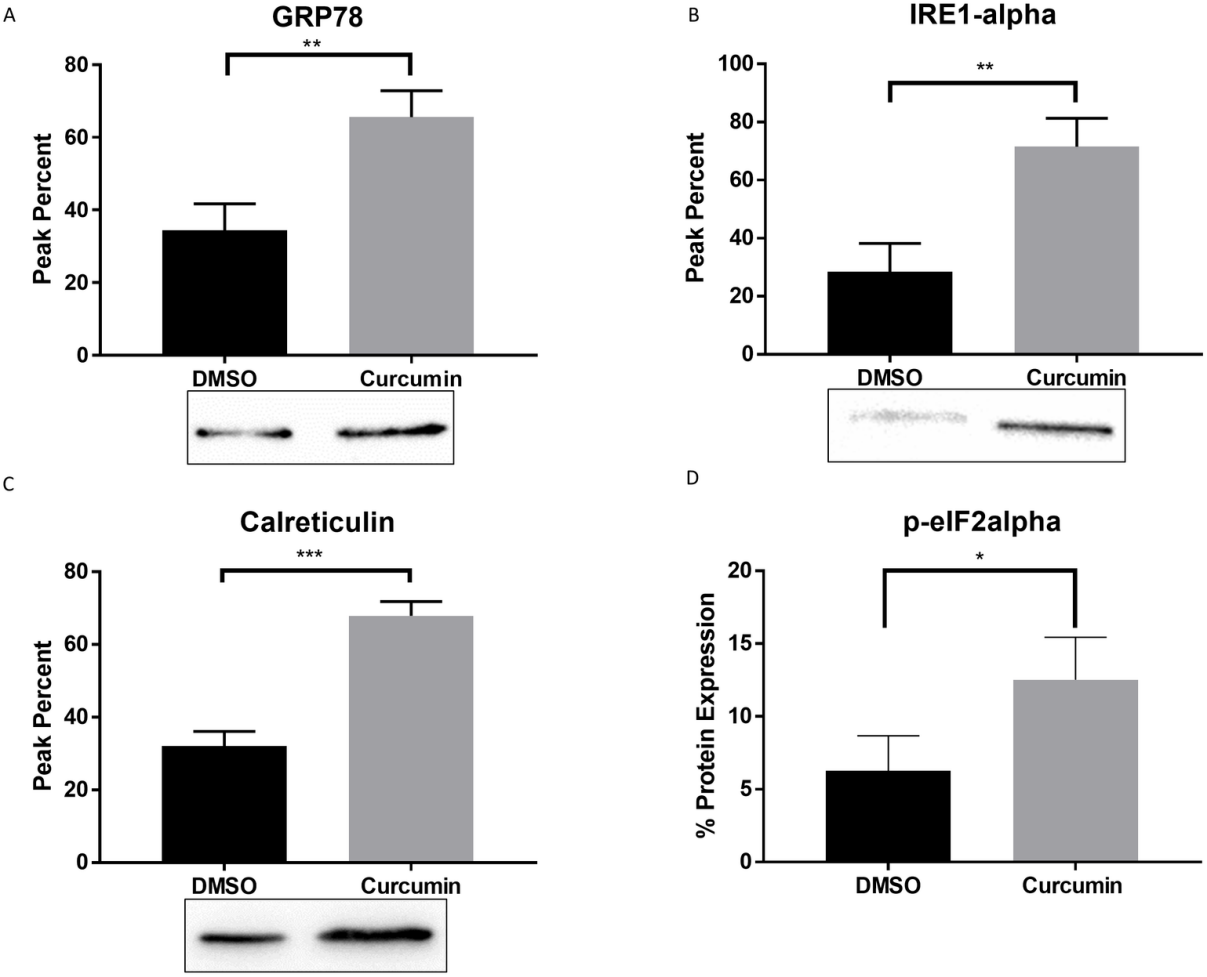
Curcumin triggers ER stress. (A-C) Protein expression of ER stress markers: GRP78, IRE-1α and Calreticulin analyzed by western blotting. (D) Phosphorylated expression of eIF2α protein assessed by flow cytometry. Statistical significance between Curcumin and DMSO was determined by unpaired t-test, * p≤0.05, ** p≤0.01, ***p≤0.001.

In order to validate the ER stress curcumin-induced protein alterations obtained by the quantitative proteomics results (Table 1), cell extracts from curcumin-treated cells were analyzed by flow cytometry and western blotting. As depicted in Fig 3, curcumin treatment resulted in the upregulation of common ER stress markers GRP78, inositol-requiring enzyme 1 (Ire1α) and calreticulin (Fig 3A–3C, p value ≤ 0.0064, 0.0056 and 0.0004 respectively). Expression of p-eIF2α, a regulator in the transient translation attenuation and in the induction of apoptosis, was evaluated through flow cytometry analysis. Curcumin induced the expression of p-eIF2α by nearly 12% when compared to 6% in DMSO (Fig 3D, p value ≤ 0.0455). These results suggest that curcumin may be inducing cell death through the ER stress upstream PERK/eIF2α/ATF4 pathway in PC3 cells.

**Fig 4:**
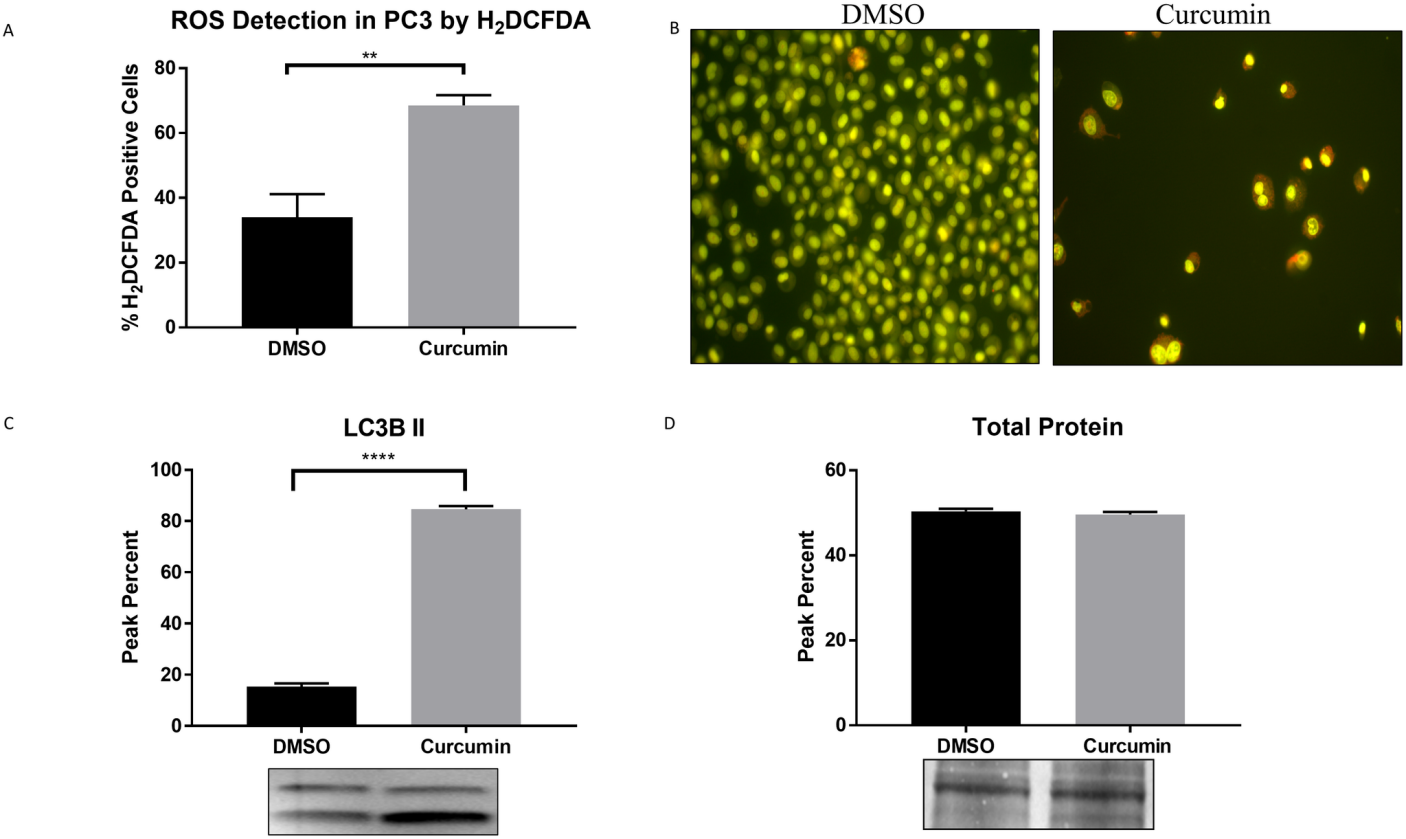
Curcumin promotes oxidative stress and autophagy. (A) Increased production of ROS was measured using the H_2_DCFDA technique by flow cytometry. (B) Acridine orange fluorescence micrographs in curcumin and DMSO treated PC3 cells. (C) LC3B II protein expression analyzed by western blot. D. Total protein expression by india ink staining. Statistical significance between Curcumin and DMSO was determined by unpaired t-test, ** p≤0.01, **** p≤0.0001.

Evidences suggest that ER stress can play a key role in autophagy regulation and oxidative stress [13, 22]. To further validate the quantitative proteomics results (Table 1), curcumin’s role in promoting cell death mediated chronic ER/oxidative stresses and autophagy responses were evaluated. Upon curcumin treatment, cells were exposed to 1μM of HD2CFDA for 30 minutes to determine intracellular oxidation levels. PC3 cells treated with curcumin had nearly 70% of HD2CFDA positive cells when compared to approximately 35% cells treated with DMSO, with a p value ≤ 0.0016 (Fig 4A).

It is generally accepted that ROS induce autophagy [23], considered as a tumor suppressor mechanism by preventing ROS accumulation through elimination of damaged mitochondria. However, under prolonged or severe ER stress, apoptosis can be prompted by autophagy. To evaluate if curcumin induces autophagy, PC3 cells were treated with 5μg/mL curcumin for 72 hours and stained with acridine orange (Fig 4B). Induction of autophagy by curcumin was indicated by increased acridine orange staining due to lysosomal acidity. To confirm curcumin-induced autophagic activity, western blotting technique was used by assessing LC3B expression, a common autophagic marker. An increased level of LC3B expression with a peak percent in the range of over 80% was detected in curcumin treated PC3 cells when compared to approximately 15% in DMSO (Fig 4C, p value ≤ 0.0001).

Overall, these findings, together with the results described above, correlate with the upregulation of pro-apoptotic markers PARP and caspases 3, 9 and 12, that in turn support this study hypothesis, indicating that curcumin induces a chronic ER stress mediated cell death and activates cell cycle arrest, UPR, autophagy and oxidative stress responses.

## Discussion

Anti-cancer therapies, such as chemotherapy and radiation, among others, have been shown to be aggressive approaches for treating cancer. In this study, nearly 50% of cells treated with 5 μg/ml curcumin lost viability in highly metastatic prostate cancer cells (PC3). Curcumin inhibited the growth of PC3 cells and induced a chronic ER stress and oxidative species dependent responses promoting cell death. Accumulation of misfolded proteins in the ER can disrupt ER function resulting in ER stress. The Unfolded Protein Response (UPR) functions as a cytoprotective mechanism aimed at decreasing the levels of unfolded proteins and coordinating the induction of ER chaperones with decreased protein synthesis and cell cycle growth arrest in the G1 phase. UPR activation of PERK may result in translational repression of cyclin D1, a protein required for cell cycle progression through the G1 phase, via eIF2α phosphorylation [24]. The cell cycle analysis depicted in Fig 1C revealed that curcumin caused a G0/G1 phase cell cycle arrest in PC3. Furthermore, the isobaric TMT labeling-based proteomic quantification revealed that ERp29 was overexpressed in curcumin treated cells. ERp29 is linked to PERK activation and eIF2α phosphorylation resulting in cell cycle inhibition and tumor suppression [25]. Not surprisingly, quantitative proteomic results indicate that CDK1, a key regulator of cell division, migration and inducer of mitotic cell cycle progression, was downregulated to possibly prevent cell proliferation and promote apoptosis [26, 27]. Moreover, PPP1CA, regulator of cell cycle progression and proliferation, was downregulated by -1.35-fold in curcumin treated cells [28, 29]. Thus, our results suggest that curcumin induced an UPR prompting G0/G1 cell cycle arrest.

Recent developments in the literature have demonstrated that the UPR is an important mechanism required for cancer cells to maintain malignancy. Cancer cells can adapt to ER stress and evade stress-induced apoptotic pathways by activating the UPR branches [30]. During acute ER stress, chaperone proteins are activated to correct misfolding and re-establish cellular homeostasis through the activation of the ER-associated degradation pathway. On the other hand, chronic ER stress causes UPR-mediated apoptosis. Data obtained from the quantitative proteomics TMT analysis were consistent with the cytotoxic evidence, demonstrating a strong correlation between ER stress and apoptotic markers. The proteomic data reveals that GRP78, a key ER stress marker, was upregulated in PC3 curcumin treated cells. GRP78 is a resident ER protein responsible for the regulation of the proper refolding of misfolded proteins [31]. The importance of this chaperone is directly linked to activation of the three transmembrane UPR signaling initiators: PERK, IRE1, and activating transcription factor 6. Another ER stress chaperone marker, PDI, was significantly upregulated at a fold change of 1.25 after curcumin treatment and as identified by TMT quantitative proteomics data. The PDI superfamily can act as molecular chaperones, protein-binding partners, hormone reservoirs and recently, have been implicated in the apoptotic signaling pathways [32]. Other important ER stress markers, such as calreticulin were validated through western blot analysis. This chaperone is involved in protein folding and Ca^2+^ homeostasis in the ER and can act as a proapoptotic protein under stress conditions [33]. If homeostasis cannot be reinstated by the previously mentioned pathways, the cells resort to activation of apoptosis. Another indispensable pathway involved in cellular homeostasis is the PERK–eIF2α signaling. Phosphorylated eIF2α was significantly upregulated when compared with control conditions. Upon UPR activation, eIF2α is phosphorylated by PERK to produce signaling that halts the translation of proteins in order to prevent misfolded proteins accumulation. The upregulation of eIF2α in curcumin-treated cells, suggests activation of the UPR-associated PERK pathway. Additional evidence for UPR activation is the deregulation of proteasomal proteins PMPCA, PSMB2, PSMC1 and PSMD2, demonstrating ERAD activation [34]. Under chronic ER stress settings, PERK phosphorylates eIF2α leading to the expression of transcription factor ATF4 through the PERK-eIF2α pathway. ATF4 induces the transcription of C/EBP homologous protein (CHOP, also known as GADD153) subsequently activating caspase 12 [21]. Given the important role of caspases as effector molecules in various forms of cell death including curcumin-induced apoptosis, the ability of these anti-cancer agents to trigger caspase activation was evaluated. Caspase 12 upregulation appears to be a critical determinant of curcumin cytotoxic effect on PCa, since caspase 12 activation is an indicator of ER-stress-induced apoptosis. As extensively discussed in the literature, upon activation, caspase 12 may cleave caspase 9, which in turn activates the effector caspase 3 [35]. Caspase 3 may then cleave poly (ADP-ribose) polymerase-1 (PARP-1), involved in DNA damage and induction of programmed cell death [36]. Our results showed significantly increased expression of nearly all the proteins involved in PERK pathway such as p-eIF2α, caspase 12, 9, 3 and PARP, indicating that curcumin induced apoptosis via ER-stress. Another master regulator in cell fate determination under ER stress, IRE1, is known to initiate apoptosis via splicing of the transcription factor XBP1 and through Regulated IRE1-Dependent Decay (RIDD) activation [37]. IRE1α upregulation, supports the hypothesis that curcumin induces ER stress mediated cell death in PC3.

Likewise, the downregulation of anti-apoptotic proteins (inorganic pyrophosphatase (PPA1) and vesicle-associated membrane protein-associated protein A or VAPA) and promoting pro-apoptotic proteins, such as annexin 5 and PGRMC1 similarly lead to cell death. PPA1 enzyme has been implicated in cell survival and is essential for controlling cellular levels of inorganic pyrophosphate. Moreover, it has been proven that PPA1 silencing significantly increase cell apoptosis [38]; while, VAPA has been demonstrated to protect cells against stress-induced apoptosis. On the other hand, pro-apoptotic annexin 5, localized in the mitochondrial outer membrane, may undergo a conformational change to activate caspase 3 signaling, leading to apoptosis. Based on the quantitative proteomic data, nearly all cytoskeletal proteins (tubulin, profilin, myosin, dynamin and destrin) were downregulated in curcumin treated PC3 cells, possibly during apoptosis as demonstrated by previous evidences [39, 40]. Our findings reaffirm that the cytoskeletal reorganization plays a crucial role in cell homeostasis maintenance and execution of cell stress responses. Protein folding and chaperones ensure that only properly folded proteins exit the ER compartment. Consequently, ER calcium, alteration in the redox status or generation of ROS, and energy deprivation can affect protein folding mechanisms and ER homeostasis [41]. Both ER and oxidative stresses, through ROS generation, may prompt apoptosis under chronic stress conditions. Table 1 showed significant upregulation of oxidative stress regulators sulfiredoxin, PDZ and LIM domain protein 1, while peroxiredoxins and DJ-1 (PARK7) proteins known to lessen oxidative damage, were downregulated. Studies demonstrate that these two proteins act as protective mediators to prevent ROS-induced apoptosis[42, 43]. Imbalances between ROS and antioxidant molecules, however, result in oxidative stress. Malignant cells depend on these antioxidant mechanisms for survival as they transform, and their oncogenic activity rely on their ability to increase a cell’s resistance to ROS [44]. Intracellular ROS detection by H2DCFDA showed an increased activity of oxidative species in curcumin treated cells when compared to DMSO, confirming that curcumin induced ROS and contributed to oxidative stress. Our findings establish that ROS played as an activator of cell death, since increased levels of ROS were observed while antioxidants expression was downregulated in curcumin treated cells. Subsequently, oxidant species accumulation can contribute to irreversible damage to biomolecules leading to apoptosis. These results suggest that curcumin orchestrates an apoptotic mechanism against prostate cancer cells by an ER and oxidative stress-induced apoptosis.

Autophagy, a major catabolic process which degrades and recycles proteins by engulfing autophagic substrates, can be triggered by the UPR as misfolded proteins and ROS accumulate [10]. Elevated levels of ROS activate PERK-eIF2α pathway for autophagy induction [45]. Additionally, oxidation modification results in the formation of LC3-associated autophagosomes. Curcumin induced increased expression of the LC3B autophagic marker when compared to DMSO treated cells. Moreover, Rab GDP dissociation inhibitor beta (GDI2), general regulator for the membrane association of RAB proteins, was downregulated. Similar to previous observations, deregulation of GDI2 affects the activation of RAB protein, key protagonists in autophagosome formation and maturation [28, 46]. Results obtained with acridine orange staining further validate curcumin’s effect on the increased autophagic activity. Cells deficient in DJ-1 (PARK7), a protein known to act in the regulation of mitochondrial function and autophagy have been shown to increase LC3 puncta and to induce higher levels of ROS [47]. Table 1 reveal that curcumin treated cells showed a DJ-1 deficiency. This could lead to multiple mitochondrial abnormalities, including decreased membrane potential, fragmented mitochondria and reduced connectivity, consequences of oxidative stress [47].

**Fig 5:**
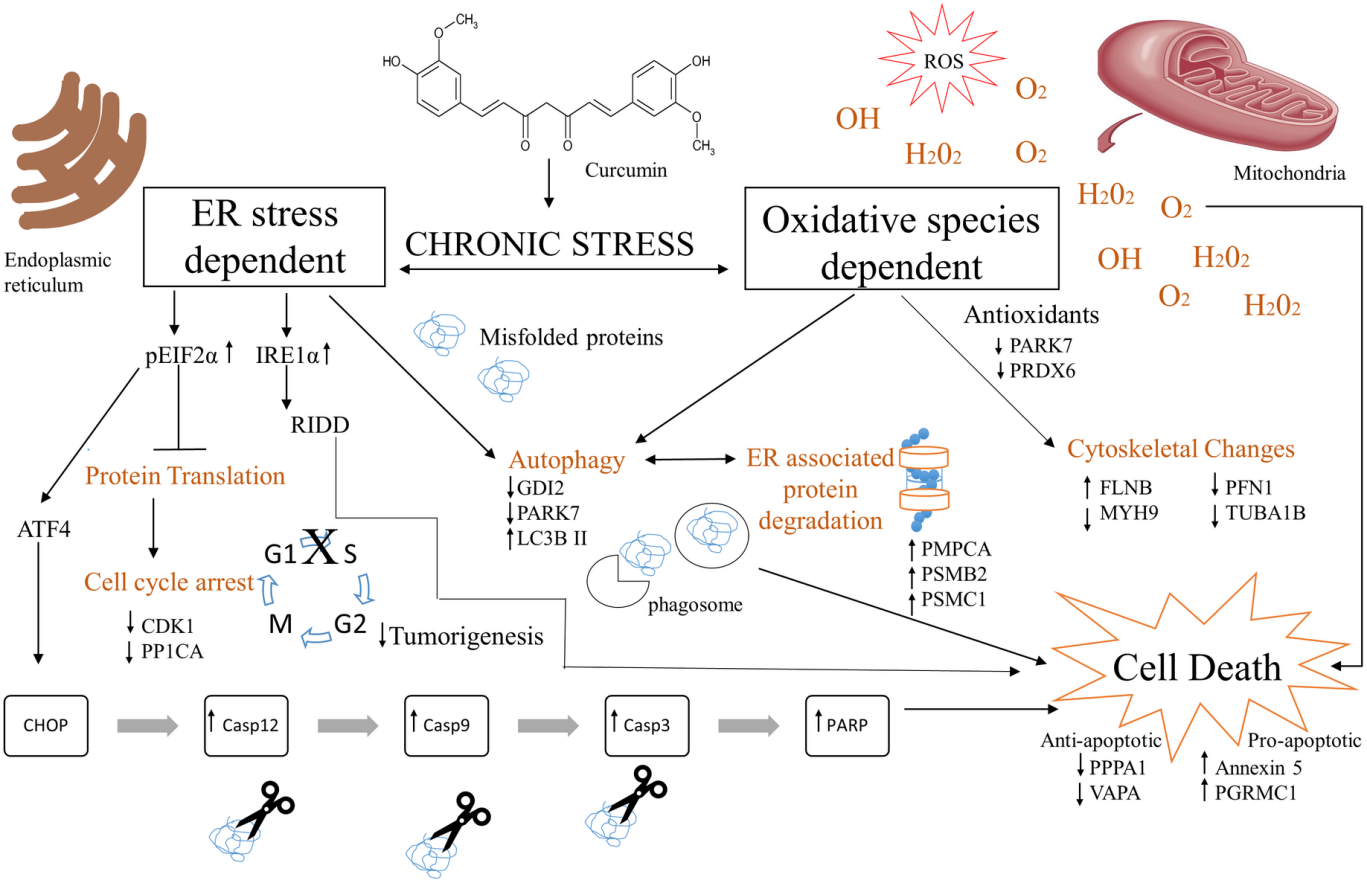
Proposed molecular mechanism of Curcumin’s cytotoxic effects in metastatic prostate cancer cells (PC3). Curcumin causes a chronic ER and oxidative stress inducing autophagy, cell cycle arrest, ERAD, cytoskeletal changes and caspase mediated apoptosis. Upward (↑) and downward (↓) arrows indicate upregulation or downregulation of proteins respectively.

The cross-talk of ER and oxidative stress autophagic mediated apoptotic signaling observed in curcumin treated PC3 cells is shown in Fig 5. This proposed model highlights the differentially expressed proteins identified by the quantitative TMT isobaric labeling proteomic analysis and validated by qRT-PCR, western blot and flow cytometry. The upregulated proteins (upward arrows) were shown to be involved mainly in ER and oxidative stress mechanisms, autophagy, cell cycle arrest and apoptosis in curcumin treated cells. Furthermore, the results obtained suggest that ER stress proteins GRP78, PDI, calreticulin together with a set of chaperones, played a key role to activate and prompt this pro-apoptotic mechanism in PC3 cells. Moreover, curcumin induced a caspase dependent pro-apoptotic response as validated by qRT-PCR analysis. Downregulation of apoptotic proteins (downward arrows) support the anti-apoptotic/survival response obtained in DMSO treated PC3 cells. Collectively, these data provide a plausible mechanistic rationale for curcumin role in modulating major targeted cell signaling pathways subsequently activating cell death signals inducing apoptosis in prostate cancer cells.

To our knowledge, this study is the first integrated gel-free shotgun quantitative proteomic mass spectrometry-based analysis coupled with Tandem Mass Tag isobaric labeling-based-signaling on the effect of curcumin in prostate cancer. Curcumin acted as a potent cancer growth suppressive and cytotoxic agent in PC3, via the impairment of multiple signaling mechanisms, including chronic ER stress mediated pro-apoptotic pathways. Our dataset provide insight into targets and mechanistic strategies of the anti-cancer effect of curcumin and will serve as a useful resource for future studies.

## Supporting information

S1 Table All proteins identified by TMT in curcumin and DMSO treated PC3 cells. (XLSX)

## References

[1] Siegel RL, Miller KD, Jemal A. Cancer statistics, 2016. CA: a cancer journal for clinicians. 2016;66(1):7–30. Epub 01/09. doi: 10.3322/caac.21332 26742998 10.3322/caac.21332

[2] Plenderleith IH. Treating the treatment: toxicity of cancer chemotherapy. Can Fam Physician. 1990;36:1827–30. ; PubMed Central PMCID: PMCPMC2280515. 21234006 PMC2280515

[3] Wu SH, Hang LW, Yang JS, Chen HY, Lin HY, Chiang JH, et al Curcumin induces apoptosis in human non-small cell lung cancer NCI-H460 cells through ER stress and caspase cascade- and mitochondria-dependent pathways. Anticancer Res. 2010;30(6):2125–33. . 20651361

[4] Zheng R, Deng Q, Liu Y, Zhao P. Curcumin Inhibits Gastric Carcinoma Cell Growth and Induces Apoptosis by Suppressing the Wnt/beta-Catenin Signaling Pathway. Med Sci Monit. 2017;23:163–71. doi: 10.12659/MSM.902711 28077837 10.12659/MSM.902711 PMC5248567

[5] Senft C, Polacin M, Priester M, Seifert V, Kogel D, Weissenberger J. The nontoxic natural compound Curcumin exerts anti-proliferative, anti-migratory, and anti-invasive properties against malignant gliomas. BMC Cancer. 2010;10:491 ; PubMed Central PMCID: PMCPMC2949804. doi: 10.1186/1471-2407-10-491 20840775 10.1186/1471-2407-10-491 PMC2949804

[6] Sundram V, Chauhan SC, Ebeling M, Jaggi M. Curcumin attenuates beta-catenin signaling in prostate cancer cells through activation of protein kinase D1. PLoS One. 2012;7(4):e35368 ; PubMed Central PMCID: PMCPMC3327669. doi: 10.1371/journal.pone.0035368 22523587 10.1371/journal.pone.0035368 PMC3327669

[7] Menon VP, Sudheer AR. Antioxidant and anti-inflammatory properties of curcumin. Adv Exp Med Biol. 2007;595:105–25. doi: 10.1007/978-0-387-46401-5_3 17569207 10.1007/978-0-387-46401-5_3

[8] Bakhshi J, Weinstein L, Poksay KS, Nishinaga B, Bredesen DE, Rao RV. Coupling endoplasmic reticulum stress to the cell death program in mouse melanoma cells: effect of curcumin. Apoptosis. 2008;13(7):904–14. ; PubMed Central PMCID: PMCPMC2727483. doi: 10.1007/s10495-008-0221-x 18493855 10.1007/s10495-008-0221-x PMC2727483

[9] Zhao L, Ackerman SL. Endoplasmic reticulum stress in health and disease. Curr Opin Cell Biol. 2006;18(4):444–52. doi: 10.1016/j.ceb.2006.06.005 16781856 10.1016/j.ceb.2006.06.005

[10] Senft D, Ronai ZA. UPR, autophagy, and mitochondria crosstalk underlies the ER stress response. Trends Biochem Sci. 2015;40(3):141–8. ; PubMed Central PMCID: PMCPMC4340752. doi: 10.1016/j.tibs.2015.01.002 25656104 10.1016/j.tibs.2015.01.002 PMC4340752

[11] Yonekawa T, Thorburn A. Autophagy and Cell Death. Essays Biochem. 2013;55:105–17. doi: 10.1042/bse0550105 24070475 10.1042/bse0550105 PMC3894632

[12] Cao SS, Kaufman RJ. Endoplasmic reticulum stress and oxidative stress in cell fate decision and human disease. Antioxid Redox Signal. 2014;21(3):396–413. ; PubMed Central PMCID: PMCPMC4076992. doi: 10.1089/ars.2014.5851 24702237 10.1089/ars.2014.5851 PMC4076992

[13] Malhotra JD, Kaufman RJ. Endoplasmic reticulum stress and oxidative stress: a vicious cycle or a double-edged sword? Antioxid Redox Signal. 2007;9(12):2277–93. doi: 10.1089/ars.2007.1782 17979528 10.1089/ars.2007.1782

[14] Sokka AL, Putkonen N, Mudo G, Pryazhnikov E, Reijonen S, Khiroug L, et al Endoplasmic reticulum stress inhibition protects against excitotoxic neuronal injury in the rat brain. J Neurosci. 2007;27(4):901–8. doi: 10.1523/JNEUROSCI.4289-06.2007 17251432 10.1523/JNEUROSCI.4289-06.2007 PMC6672923

[15] Eskelinen EL. The dual role of autophagy in cancer. Curr Opin Pharmacol. 2011;11(4):294–300. doi: 10.1016/j.coph.2011.03.009 21498118 10.1016/j.coph.2011.03.009

[16] Kurapati KR, Samikkannu T, Kadiyala DB, Zainulabedin SM, Gandhi N, Sathaye SS, et al Combinatorial cytotoxic effects of Curcuma longa and Zingiber officinale on the PC-3M prostate cancer cell line. J Basic Clin Physiol Pharmacol. 2012;23(4):139–46. ; PubMed Central PMCID: PMCPMC4561547. doi: 10.1515/jbcpp-2012-0021 23072849 10.1515/jbcpp-2012-0021 PMC4561547

[17] Rao KVK, Boukli NM, Samikkannu T, Cubano LA, Dakshayani BK, Nair M P.N.. Proteomics Profiling and Cytotoxic Effect of Curcuma longa on Prostate Cancer. The Open Proteomics Journal. 2011;4:1–11.

[18] Burton LJ, Rivera M, Hawsawi O, Zou J, Hudson T, Wang G, et al Muscadine Grape Skin Extract Induces an Unfolded Protein Response-Mediated Autophagy in Prostate Cancer Cells: A TMT-Based Quantitative Proteomic Analysis. PLoS One. 2016;11(10):e0164115 ; PubMed Central PMCID: PMCPMC5068743. doi: 10.1371/journal.pone.0164115 27755556 10.1371/journal.pone.0164115 PMC5068743

[19] Ryan D, Carberry S, Murphy AC, Lindner AU, Fay J, Hector S, et al Calnexin, an ER-induced protein, is a prognostic marker and potential therapeutic target in colorectal cancer. J Transl Med. 2016;14(1):196 ; PubMed Central PMCID: PMCPMC4930591. doi: 10.1186/s12967-016-0948-z 27369741 10.1186/s12967-016-0948-z PMC4930591

[20] Rhee HJ, Kim GY, Huh JW, Kim SW, Na DS. Annexin I is a stress protein induced by heat, oxidative stress and a sulfhydryl-reactive agent. Eur J Biochem. 2000;267(11):3220–5. Epub 05/29. . 10824106 10.1046/j.1432-1327.2000.01345.x

[21] Nakagawa T, Zhu H, Morishima N, Li E, Xu J, Yankner BA, et al Caspase-12 mediates endoplasmic-reticulum-specific apoptosis and cytotoxicity by amyloid-beta. Nature. 2000;403(6765):98–103. doi: 10.1038/47513 10638761 10.1038/47513

[22] Yan MM, Ni JD, Song D, Ding M, Huang J. Interplay between unfolded protein response and autophagy promotes tumor drug resistance. Oncol Lett. 2015;10(4):1959–69. doi: 10.3892/ol.2015.3508 26622781 10.3892/ol.2015.3508 PMC4579870

[23] Azad MB, Chen Y, Gibson SB. Regulation of autophagy by reactive oxygen species (ROS): implications for cancer progression and treatment. Antioxid Redox Signal. 2009;11(4):777–90. doi: 10.1089/ARS.2008.2270 18828708 10.1089/ars.2008.2270

[24] Brewer JW, Diehl JA. PERK mediates cell-cycle exit during the mammalian unfolded protein response. Proc Natl Acad Sci U S A. 2000;97(23):12625–30. ; PubMed Central PMCID: PMCPMC18814. doi: 10.1073/pnas.220247197 11035797 10.1073/pnas.220247197 PMC18814

[25] Chen S, Zhang D. Friend or foe: Endoplasmic reticulum protein 29 (ERp29) in epithelial cancer. FEBS open bio. 2015;5:91–8. Epub 02/25. doi: 10.1016/j.fob.2015.01.004 25709888 10.1016/j.fob.2015.01.004 PMC4329646

[26] Barbacid DS, Cédric B, Antonio C, Sarah H, Claudine T, Kathryn N, et al Cdk1 is sufficient to drive the mammalian cell cycle. Nature. 2007;448(7155):811–5. doi: 10.1038/nature06046 17700700 10.1038/nature06046

[27] Kaldis MKD, Chandrahas Koumar R, Padmakumar VC, Tiehua D, Martin W, Vincenzo C, et al Cyclin-dependent kinase 1 (Cdk1) is essential for cell division and suppression of DNA re-replication but not for liver regeneration. 2012 10.1073/pnas.1115201109 10.1073/pnas.1115201109 PMC3309725 22355113

[28] Ao X, Zou L, Wu Y. Regulation of autophagy by the Rab GTPase network. Cell Death Differ. 2014;21(3):348–58. ; PubMed Central PMCID: PMCPMC3921601. doi: 10.1038/cdd.2013.187 24440914 10.1038/cdd.2013.187 PMC3921601

[29] Trinkle-Mulcahy L, Lamond AI. Mitotic phosphatases: no longer silent partners. Curr Opin Cell Biol. 2006;18(6):623–31. doi: 10.1016/j.ceb.2006.09.001 17030123 10.1016/j.ceb.2006.09.001

[30] Wang G, Yang ZQ, Zhang K. Endoplasmic reticulum stress response in cancer: molecular mechanism and therapeutic potential. Am J Transl Res. 2010;2(1):65–74. ; PubMed Central PMCID: PMCPMC2826823. 20182583 PMC2826823

[31] Li J, Lee AS. Stress induction of GRP78/BiP and its role in cancer. Curr Mol Med. 2006;6(1):45–54. . 16472112 10.2174/156652406775574523

[32] Grek C, Townsend DM. Protein Disulfide Isomerase Superfamily in Disease and the Regulation of Apoptosis. Endoplasmic Reticulum Stress Dis. 2014;1(1):4–17. ; PubMed Central PMCID: PMCPMC4192724. doi: 10.2478/ersc-2013-0001 25309899 10.2478/ersc-2013-0001 PMC4192724

[33] Lopez Sambrooks C, Carpio MA, Hallak ME. Arginylated calreticulin at plasma membrane increases susceptibility of cells to apoptosis. J Biol Chem. 2012;287(26):22043–54. Epub 05/12. doi: 10.1074/jbc.M111.338335 22577148 10.1074/jbc.M111.338335 PMC3381163

[34] Gomes AV. Genetics of proteasome diseases. Scientifica (Cairo). 2013;2013:637629 ; PubMed Central PMCID: PMCPMC3892944. doi: 10.1155/2013/637629 24490108 10.1155/2013/637629 PMC3892944

[35] Szegezdi E, Fitzgerald U, Samali A. Caspase-12 and ER-stress-mediated apoptosis: the story so far. Ann N Y Acad Sci. 2003;1010:186–94. . 15033718 10.1196/annals.1299.032

[36] Los M, Mozoluk M, Ferrari D, Stepczynska A, Stroh C, Renz A, et al Activation and caspase-mediated inhibition of PARP: a molecular switch between fibroblast necrosis and apoptosis in death receptor signaling. Mol Biol Cell. 2002;13(3):978–88. ; PubMed Central PMCID: PMCPMC99613. doi: 10.1091/mbc.01-05-0272 11907276 10.1091/mbc.01-05-0272 PMC99613

[37] Chen Y, Brandizzi F. IRE1: ER stress sensor and cell fate executor. Trends Cell Biol. 2013;23(11):547–55. ; PubMed Central PMCID: PMCPMC3818365. doi: 10.1016/j.tcb.2013.06.005 23880584 10.1016/j.tcb.2013.06.005 PMC3818365

[38] Lin HY, Yin Y, Zhang JX, Xuan H, Zheng Y, Zhan SS, et al Identification of direct forkhead box O1 targets involved in palmitate-induced apoptosis in clonal insulin-secreting cells using chromatin immunoprecipitation coupled to DNA selection and ligation. Diabetologia. 2012;55(10):2703–12. doi: 10.1007/s00125-012-2643-9 22810813 10.1007/s00125-012-2643-9

[39] Grzanka A, Grzanka D, Orlikowska M. Cytoskeletal reorganization during process of apoptosis induced by cytostatic drugs in K-562 and HL-60 leukemia cell lines. Biochem Pharmacol. 2003;66(8):1611–7. . 14555241 10.1016/s0006-2952(03)00532-x

[40] Parker AL, Kavallaris M, McCarroll JA. Microtubules and Their Role in Cellular Stress in Cancer. Front Oncol. 2014;4 doi: 10.3389/fonc.2014.00153 24995158 10.3389/fonc.2014.00153 PMC4061531

[41] Zeeshan HM, Lee GH, Kim HR, Chae HJ. Endoplasmic Reticulum Stress and Associated ROS. Int J Mol Sci. 2016;17(3):327 ; PubMed Central PMCID: PMCPMC4813189. doi: 10.3390/ijms17030327 26950115 10.3390/ijms17030327 PMC4813189

[42] Whitaker HC, Patel D, Howat WJ, Warren AY, Kay JD, Sangan T, et al Peroxiredoxin-3 is overexpressed in prostate cancer and promotes cancer cell survival by protecting cells from oxidative stress. Br J Cancer. 2013;109(4):983–93. ; PubMed Central PMCID: PMCPMC3749568. doi: 10.1038/bjc.2013.396 23880827 10.1038/bjc.2013.396 PMC3749568

[43] Kawate T, Iwaya K, Kikuchi R, Kaise H, Oda M, Sato E, et al DJ-1 protein expression as a predictor of pathological complete remission after neoadjuvant chemotherapy in breast cancer patients. Breast Cancer Res Treat. 2013;139(1):51–9. doi: 10.1007/s10549-013-2523-0 23592371 10.1007/s10549-013-2523-0

[44] Kim DK, Beaven MA, Kulinski JM, Desai A, Bandara G, Bai Y, et al Regulation of Reactive Oxygen Species and the Antioxidant Protein DJ-1 in Mastocytosis. PLoS One. 2016;11(9):e0162831 ; PubMed Central PMCID: PMCPMC5017616. doi: 10.1371/journal.pone.0162831 27611333 10.1371/journal.pone.0162831 PMC5017616

[45] Poillet-Perez L, Despouy G, Delage-Mourroux R, Boyer-Guittaut M. Interplay between ROS and autophagy in cancer cells, from tumor initiation to cancer therapy. Redox biology. 2015;4:184–92. Epub 01/16. doi: 10.1016/j.redox.2014.12.003 25590798 10.1016/j.redox.2014.12.003 PMC4803791

[46] Ullrich O, Stenmark H, Alexandrov K, Huber LA, Kaibuchi K, Sasaki T, et al Rab GDP dissociation inhibitor as a general regulator for the membrane association of rab proteins. J Biol Chem. 1993;268(24):18143–50. . 8349690

[47] McCoy MK, Cookson MR. DJ-1 regulation of mitochondrial function and autophagy through oxidative stress. Autophagy. 2011;7(5):531–2. ; PubMed Central PMCID: PMCPMC3127213. doi: 10.4161/auto.7.5.14684 21317550 10.4161/auto.7.5.14684 PMC3127213

